# Identification of potential SARS‐CoV‐2 genomic regions representing hallmarks for adaptation to different hosts

**DOI:** 10.1002/imo2.70019

**Published:** 2025-05-02

**Authors:** Janusz Wiśniewski, Heng‐Chang Chen

**Affiliations:** ^1^ Quantitative Virology Research Group, Population Diagnostics Center Łukasiewicz Research Network – PORT Polish Center for Technology Development Wrocław Poland; ^2^ The Laboratory of Quantitative Virology Centre for Advanced Materials and Technologies, Warsaw University of Technology Warsaw Poland

**Keywords:** host adaptation, K‐mers, SARS‐CoV‐2

## Abstract

The *k*‐mer‐based pipeline, namely the Pathogen Origin Recognition Tool using Enriched *K*‐mers (PORT‐EK) identifies genomic regions enriched in the respective hosts after the comparison of multi‐genomes of isolates between different host species. The enriched *k*‐mer counts, which may serve as a potential marker, enable the classification and prediction of the likelihood of the host species. Altogether, PORT‐EK showcased its feasibility for identifying viral genomic regions over‐represented in respective hosts, illuminating the different intrinsic tropisms of coronavirus host adaptation.

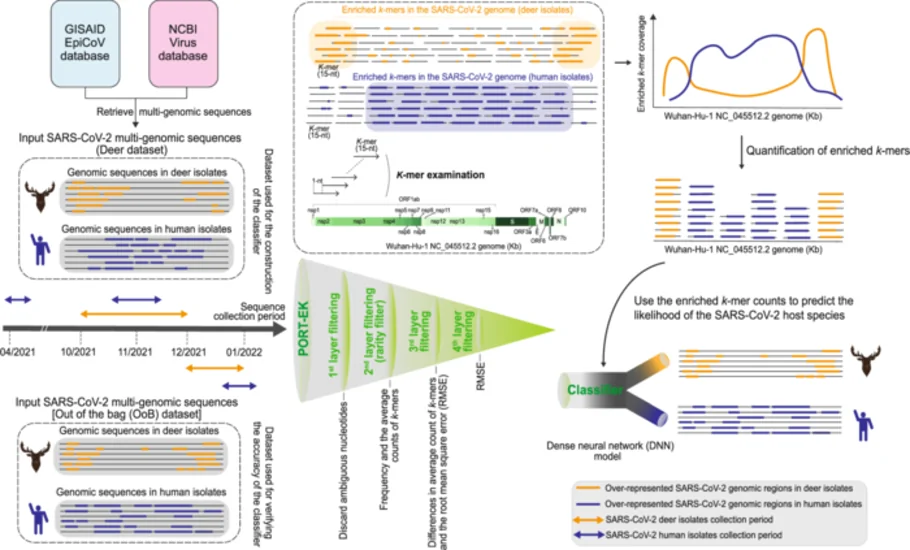

To the editor,

After the outbreak of the coronavirus disease 2019 (COVID‐19) pandemic, a significant number of severe acute respiratory syndrome coronavirus 2 (SARS‐CoV‐2) variant sequences are generated on a daily basis; the demand for bioinformatic approaches that enable efficient digestion of such massive information is thus rapidly rising. An efficient bioinformatic tool, enabling a rapid proceeding with multi‐genomic datasets is thus a requisite.

Global dissemination of SARS‐CoV‐2 causes spillover transmission across different host species, including humans. Spillover and spillback in SARS‐CoV‐2 transmission might result in establishing a reservoir, in which novel variants emerge and are capable of adapting to new host species. Previously, such diversity has been applied for monitoring the global distribution of different clades of SARS‐CoV‐2 genomes [1] and visualizing pandemic spread [2]. Based on these findings, we attempted to test whether or not genomic diversity may serve as a potential marker, enabling the classification and prediction of SARS‐CoV‐2 variants adapting in different animal reservoirs and humans.

We developed a *k*‐mer‐based pipeline, namely the Pathogen Origin Recognition Tool using Enriched *K*‐mers (PORT‐EK), which allows for a comparison of different multi‐genomic datasets and identifies genomic regions enriched in the respective hosts after the comparison of multi‐genomes of isolates between different host species. We examined the entirety of available multi‐genomic sequences of isolates collected from US white‐tailed deer, *Odocoileus virginianus* [3], as proof‐of‐concept, and sequences involved in the genus *Betacoronavirus* [4] across 34 bat species (Table S1) while comparing them with human isolates to evaluate the feasibility of PORT‐EK. Due to text‐length limits in the main text, we provide a detailed description of the rationale implemented in PORT‐EK in the section COMPUTATIONAL METHODS in Supporting Information, enabling readers to better comprehend our pipeline.

## 1 RESULTS AND DISCUSSION

### 1.1 Acquisition of multi‐genomic sequences and the overview and benchmarking of PORT‐EK

Multi‐genomic datasets were retrieved from GISAID Initiative EpiCoV database [5] and NCBI Virus database [6] and prepared two multi‐genomic datasets: the deer (Tables S2–S4) and the bat datasets (Tables S5–S6) (see Supporting Information for a detailed description of each data set). Of note, we excluded SARS‐CoV‐2 sequences (retrieved from GISAID) with low coverage [more than 5% of ambiguous nucleotides (nt)] and applied only complete sequences (sequence size is greater than 29,000 nt) for the analysis. For viral sequences retrieved from the NCBI database, only those marked as “nucleotide complete” were analyzed. The reason for selecting US white‐tailed deer, *Odocoileus virginianus* [3, 7], as our test species is two‐fold: (1) multiple spillover transmission from humans to white‐tailed deer within a continuous time frame inside enclosed geography has been evident [3] and (2) a sufficient number of SARS‐CoV‐2 genomic sequences was available to conduct our analyses in this study. In addition, one recent study observed an accelerated evolutionary rate of SARS‐CoV‐2 in US white‐tailed deer compared with human isolates, implying that perhaps intrinsic evolutionary constraints differ between US white‐tailed deer and other host species [8]. To our knowledge, we cannot find any more complex deer datasets than the one used in this study.

Overall, we analyzed 336 SARS‐CoV‐2 genomic sequences in deer isolates compared to human isolates in April 2021 (*n* = 21,906; namely the early 2021 human group) and in November 2021 (*n* = 11,525; namely late 2021 human group), respectively. With respect to SARS‐CoV‐2 isolates in bats (*n* = 263), we analyzed their genomic sequences in contrast with those from humans between December 2019 and February 2020 (*n* = 2081) to compute enriched *k*‐mers.

The workflow of PORT‐EK consists of four main blocks, including (1) *k*‐mers matrix preparation, (2) *k*‐mers filtering and selection, (3) identification of mutations in enriched *k*‐mers, and (4) classification of the likelihood of hosts based on the enriched *k*‐mer counts (Figure S1A) (design and the principle of PORT‐EK are detailed in Supporting Information). The brief description, functionality, and optimization of key parameters, including *k*, *c*, *m*, *min*_RESM_, *m*_map_, and *l*_map_, are summarized in Table S7 and details are provided in Supporting Information and Table S8.

To benchmark PORT‐EK we performed parallel analyses using benchmarked pipelines iMOKA [9] and KmerGO [10] (Tables S9 and S10). While processing genome sequences in the bat data set, although PORT‐EK was more time‐consuming and required more peak memory, 39, 26, and zero *k*‐mers enriched in bat could be identified using PORT‐EK, iMOKA [9], and KmerGO [10], respectively (Table S9). 98.1% enriched *k*‐mers identified by PORT‐EK could be mapped to the reference genome SARS‐CoV‐2 isolate Wuhan‐Hu‐1 (NC_045512.2), whereas 92% *k*‐mers retrieved from iMOKA [9] were mappable (Table S9). In addition to classification and principal component analysis (PCA), which could be conducted by both PORT‐EK and iMOKA [9], PORT‐EK offered the *k*‐mer coverage plot (Figure S2). While processing genome sequences in the deer data set, even though a longer running time was required using PORT‐EK (1:25:16) compared with that recorded using iMOKA [9] (0:42:52) (Table S10), a less peak memory space was needed for PORT‐EK (Table S10). 100% enriched *k*‐mers retrieved from PORT‐EK were mappable, and 0% *k*‐mers retrieved from iMOKA [9] were mappable (Table S10). Overall, this comparative assessment verified the functionality of PORT‐EK for the identification of enriched *k*‐mers in the respective hosts after the comparison of multi‐genomes of isolates between different host species.

### 1.2 Identification of enriched *k*‐mers in SARS‐CoV‐2 isolates in deer and bat datasets

**FIGURE 1:**
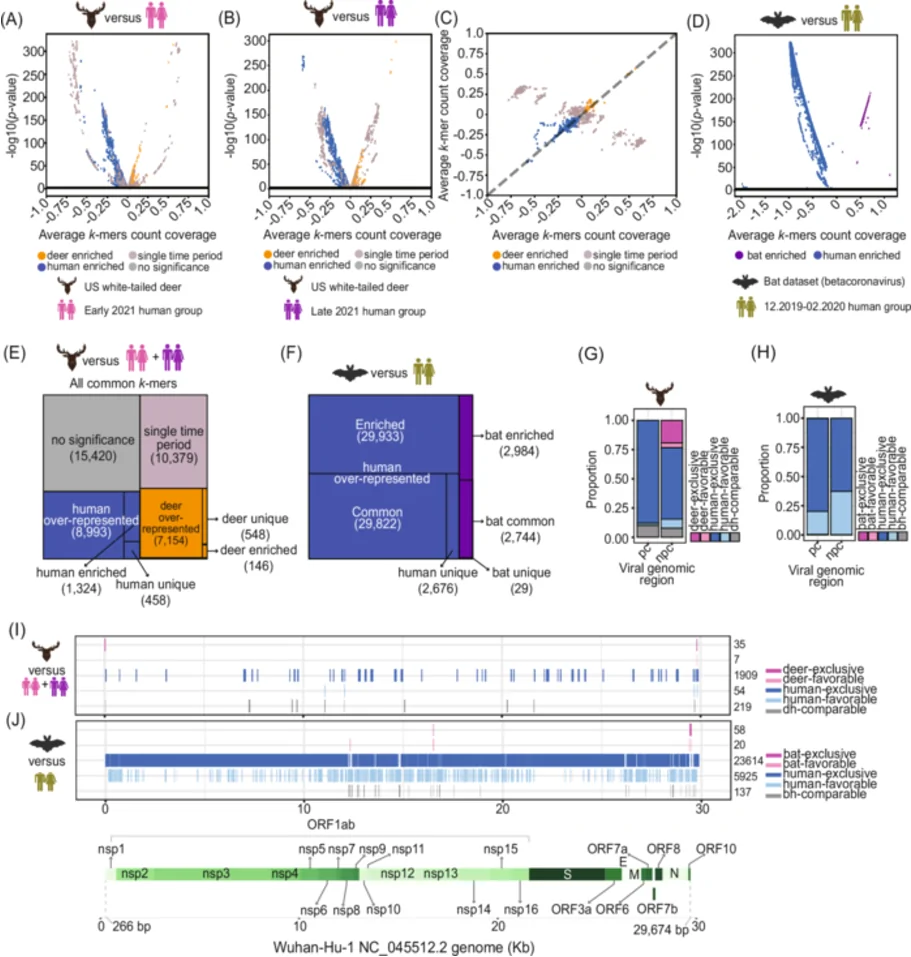
Identification of enriched *k*‐mers and landscapes of enriched *k*‐mers across the SARS‐CoV‐2 genome. Volcano plots representing *k*‐mers that were significantly over‐represented in deer isolates compared to isolates from the early (A) and late (B) 2021 human group, respectively. Each spot indicates one unique and over‐represented *k*‐mers collected after the third layer of filtering. Spots marked in orange represent *k*‐mers over‐represented in deer isolates; spots marked in blue represent *k*‐mers over‐represented in human isolates; spots marked in mauve represent *k*‐mers that were over‐represented in deer isolates against those isolated from either one of the human groups (namely, single time period); spots marked in light gray represent the rest of the *k*‐mers without significant enrichment. (C) Scatter plot specifying all subsets of *k*‐mers that were present in both Figure 1A,B, allowing the focus on over‐represented *k*‐mers generated by intrahost transmission within US white‐tailed deer. Only *k*‐mers over‐represented in deer isolates against isolates in both early and late human groups were used for further analyses. Each spot indicates one unique and significantly over‐represented *k*‐mer. Spots marked in orange represent *k*‐mers over‐represented in deer isolates; spots marked in blue represent *k*‐mers over‐represented in human isolates; spots marked in mauve represent *k*‐mers that were over‐represented in deer isolates against those isolated from either one of the human groups (namely, single time period); spots marked in light gray represent the rest of the *k*‐mers without significant enrichment. (D) Volcano plot representing enriched *k*‐mers significantly enriched in bat isolates compared to human isolates. Each spot indicates one unique and over‐represented *k*‐mers. Spots marked in purple represent *k*‐mers over‐represented in bat isolates, and spots marked in blue represent *k*‐mers over‐represented in human isolates. (E) Treemap representing the hierarchical proportion of retrieved *k*‐mers after manifesting the second layer of filtering in the deer data set. Squares marked in orange represent *k*‐mers over‐represented in deer isolates; squares marked in blue represent *k*‐mers over‐represented in human isolates; a square marked in mauve represents *k*‐mers that were over‐represented in deer isolates against those isolated from either one of the human groups (namely single time period); a square marked in light gray represents the rest of the *k*‐mers without significant enrichment. (F) Treemap representing the hierarchical proportion of retrieved *k*‐mers after manifesting the second layer of filtering in the bat data set. Squares marked in orange represent *k*‐mers over‐represented in bat isolates, and squares marked in blue represent *k*‐mers over‐represented in human isolates. (G, H) Stacked bar charts representing the proportion of enriched *k*‐mers that overlay with the protein–coding (pc) and non‐protein–coding (npc) regions throughout the SARS‐CoV‐2 genome (G, enriched *k*‐mers identified in the deer data set; H, enriched *k*‐mers identified in the bat data set). The dark pink color indicates the SARS‐CoV‐2 genomic loci overlaid exclusively with enriched *k*‐mers identified in deer (G, deer‐exclusive) or bat (H, bat‐exclusive) isolates; the light pink color indicates the loci overlaid enriched *k*‐mers present in both host species and are more quantitatively dominant in animal species (G, deer‐favorable; H, bat‐favorable); the dark blue color indicates the SARS‐CoV‐2 genomic loci overlaid exclusively with enriched *k*‐mers identified in human isolates collected in the deer (G) or bat (H) data set (namely human‐exclusive); the light blue color indicates the SARS‐CoV‐2 genomic loci overlaid enriched *k*‐mers present in both host species and are more quantitatively dominant in humans (namely human‐favorable) in the deer (G) or bat (H) data set; the gray color indicates the loci targeted by the identical number of enriched *k*‐mers between animal (deer and bat) and human isolates (namely bh‐comparable). (I, J) Line plots visualizing the genomic loci, which present overlaps with enriched *k*‐mers identified in the deer (I) and bat (J) datasets. Lines marked in the dark pink represent loci overlaid with *k*‐mers exclusively enriched in either deer (I, deer‐exclusive) or bat (J, bat‐exclusive) isolates; lines marked in light pink represent loci overlaid with *k*‐mers present in both animal (I, deer; J, bat) and human isolates and more quantitatively dominant in animals (I, deer‐favorable; J, bat‐favorable) than in humans; lines marked in the dark blue represent loci overlaid with *k*‐mers exclusively enriched in human isolates (namely human‐exclusive) in both datasets (deer, I; bats, J); lines marked in light blue represent loci overlaid with *k*‐mers present in both animal (deer, I; bats, J) and human isolates and more quantitatively dominant in humans (namely human‐favorable) than in animals; lines marked in gray represent the coverage of enriched *k*‐mers between isolates from animal hosts (deer, I; bats; J) and humans were comparable (namely bh‐comparable). Gene compositions represented by a horizontal green bar were depicted on a scale of the Wuhan‐Hu‐1 NC_045512.2 genome.

Out of a total number of 371,342 *k*‐mers, we obtained 41,946 (11.29% out of the total *k*‐mers) “common” *k*‐mers (*k*‐mers that passed through the second layer of filtering, see Supporting Information; Table S11) in the deer data set: 7154 (1.93% and 17.06% out of the total and common *k*‐mers, respectively) were over‐represented in deer isolates, 8993 (2.42% and 21.44% out of the total and common *k*‐mers, respectively) were over‐represented in human isolates, 10,379 (2.79% and 24.74% out of the total and common *k*‐mers, respectively) were over‐represented in deer isolates, against only one of the two groups of human isolates (labeled as “single time period” in Figure 1A,B), and for the remaining 15,420 (4.15% and 36.76% out of the total and common *k*‐mers, respectively), the differences were not statistically significant (Figure 1A,B,E). Of note, we focused on enriched *k*‐mers present at two independent time points in the deer data set, aiming at identifying enriched *k*‐mers resulting mainly from different host species irrespective of temporal influence (Figure 1C). Out of a total number of 1,442,011 *k*‐mers, 32,566 (2.26% out of the total *k*‐mers) common *k*‐mers were found in the bat data set: 2744 (0.19% and 8.43% out of the total and common *k*‐mers, respectively) were over‐represented in bat isolates and 29,822 (2.07% and 91.57% out of the total and common *k*‐mers, respectively) were over‐represented in human isolates (Figures 1D and 1F; Table S12). All 32,566 *k*‐mers were statistically significant (Figure 1D). After adding closely matching rare *k*‐mers (Figure S1B and detailed in Supporting Information) and the third layer of RMSE filtering, we retained 146 *k*‐mers enriched in deer isolates versus 1324 *k*‐mers enriched in human isolates (Figure 1E), with 3150 *k*‐mers enriched in bat isolates versus 29,940 *k‐*mers enriched in human isolates (Figure 1F). Overall, we verified that PORT‐EK was able to identify *k*‐mers that were enriched between different host species.

### 1.3 The intrinsic properties of the SARS‐CoV‐2 genome associated with host domestication differed between deer and bats

**FIGURE 2:**
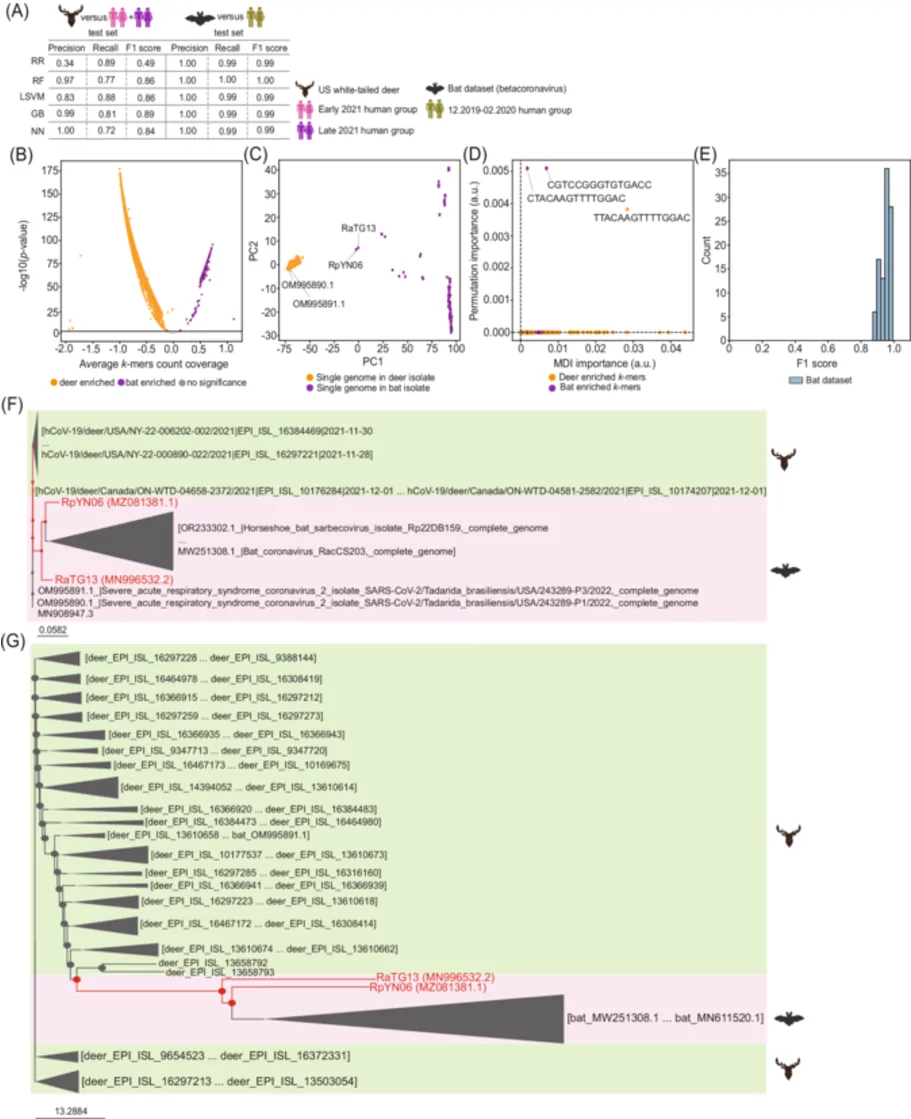
Classification and prediction of the likelihood of SARS‐CoV‐2 host species and identification of enriched *k*‐mers after the comparison of isolates between deer and bats. (A) A table recording precision, recall, and the record of F1 scores computed by two independent datasets: the deer data set (on the left‐hand side), and the bat data set (on the right‐hand side). Five models, including ridge regression classifier (RR), random forest (RF), linear support vector machine (LSVM), gradient boosting (GB), and a dense neural network (DNN) were implemented to verify the effectiveness of each model. (B) Volcano plot representing enriched *k*‐mers significantly enriched in bat isolates versus those enriched in deer isolates. Each spot indicates one enriched *k*‐mers. Spots marked in purple represent enriched *k*‐mers in bat isolates, and spots marked in orange represent enriched *k*‐mers in deer isolates; spots marked in gray represent the rest of the *k*‐mers without significant enrichment. (C) Two‐dimensional PCA revealing the discrepancy of genomic sequences of isolates collected in the deer and bats. Each dot represents a single genome of an isolate. Dots marked in orange indicate genomic sequences from deer isolates; dots marked in purple indicate genomic sequences from bat isolates. (D) Scatter plots unveiling the critical *k*‐mers for the classification of the likelihood of species between deer and bats based on the measures of the mean decrease of impurity (MDI) feature importance and permutation‐based importance. Each dot represents a single enriched *k*‐mer. Dots marked in orange indicate enriched *k*‐mers in deer; dots marked in purple indicate enriched *k*‐mers in bats. (E) Histogram representing the distribution of F1 scores measured from 100 independent train‐test splits on single genomes of isolates between deer and bats (the DNN‐based model). The distribution of F1 scores recorded from bat isolates was demonstrated after the bootstrapping on the subsets of multi‐genomes in testing datasets. Compressed versions of phylogenetic trees were constructed using either complete genomic sequences (F) or the enriched *k*‐mer count (G) between two animal species, isolated from US white‐tailed deer and bat reservoirs, consisting of 34 bat species (Table S1). Branches, branch points, and isolates written in red indicate the SARS‐CoV‐2 species, RaTG13 and RpYN06, which are two bat coronaviruses genetically close to deer SARS‐CoV‐2. Branches with a green background correspond to deer isolates, whereas branches with a pink background correspond to bat isolates. Extended versions of phylogenetic trees are provided in Figure S7.

We first observed distinct coverage landscapes of enriched *k*‐mers between the deer and bat datasets (Figure 2S). We further computed a count of SARS‐CoV‐2 genomic loci overlapping *k*‐mers and observed that 315 and 6140 genomic sites in the protein–coding regions overlaid with enriched *k*‐mers in deer (Figure 1G) and bat (Figure 1H) datasets, respectively. In the former data set, 35 SARS‐CoV‐2 genomic loci overlapped *k‐*mers exclusively enriched in deer isolates (namely, deer‐exclusive loci), 7 overlapped *k*‐mers present in deer and human isolates and more quantitatively dominant in deer than in humans (namely deer‐favorable loci), 1909 overlapped *k*‐mers exclusively enriched in human isolates (namely human‐exclusive loci), 54 overlapped *k*‐mers present in deer and human isolates and more quantitatively dominant in humans than in deer (namely human‐favorable loci), and 219 overlapped the equal number of enriched *k*‐mers in deer and human isolates (namely dh‐comparable loci) (Figure 1I). Intriguingly, deer‐exclusive and deer‐favorable sites were only detected in the non‐protein‐coding region (Figure 1G,I, and Figure S3A). A bias of the majority of human‐favorable loci overlapped the SARS‐CoV‐2 *orf10* gene (Figure 1I and Figure S3A).

In the latter data set, 58 loci overlapped *k*‐mers exclusively enriched in bat isolates (namely, bat‐exclusive loci), 20 overlapped *k*‐mers present in bats and human isolates and more quantitatively dominant in humans than in bats (namely, bat‐favorable loci), 23,614 overlapped *k*‐mers exclusively enriched in humans (namely human‐exclusive loci), 5925 overlapped *k*‐mers present in bats and human isolates and more quantitatively dominant in humans than in bats (namely human‐favorable loci), and 137 overlapped an equal number of *k*‐mers enriched in bats and humans (namely, the bh‐comparable loci) (Figure 1J). Only *k*‐mers designed to the category of human‐favorable loci unrevealed an obvious enrichment (Figures 1H,J and Figure S3A): the majority of human‐favorable sites overlaid the *orf7b* gene followed by *orf10*, *orf6*, and the gene encoding the E protein (Figure 1J and Figure S3A). A minor enrichment of *k*‐mers overlaid the *orf10* gene was observed in human‐exclusive loci and the gene encoding the N protein in bat‐exclusive and bat‐favorable loci (Figure 1J and Figure S3A). It is, however, important to note that contention about whether *orf10* is expressed during virus infection exists: multiple studies have failed to detect subgenomic RNA designated for ORF10 translation [11, 12, 13] and retaining the open reading frame may not be required for replication in vitro or in vivo [14]. Recent studies suggest that the OFR10 protein may be involved in the antiviral innate immune response [15] and contribute to more severe COVID‐19 clinical outcomes in the human host [16]. Our observation also implied the potential of the involvement of the ORF10 protein in its host adaptation. Nevertheless, in what way and how intrahost evolutionary constraints mechanistically reshape the intrinsic properties of a virus requires further investigation.

We further bootstrapped 100 (Figure S3B) and 500 (Figure S3C) enriched *k*‐mers in the deer data set and 1000 (Figure S3D) and 5000 (Figure S3E) enriched *k*‐mers in the bat data set and observed the same patterns as analyses were performed using the total number of enriched *k*‐mers (Figure S3A). Altogether, these findings signified the potential difference in the intrinsic properties of SARS‐CoV‐2 isolates between different animal hosts and humans and the feasibility of using PORT‐EK for the identification of *k*‐mers associated with specific host reservoirs.

### 1.4 The total count of enriched *k*‐mers enabled the prediction of the most probable host species of a viral genomic sequence

We selected the enriched *k*‐mers with at least one importance measure greater than zero in an attempt to acquire the minimal dimensions in the final models and subjected them to the following models: ridge regression (RR), random forest (RF), linear support vector machine, gradient boosting, and dense neural network (DNN) (Table S13). Details of the model architecture are described in Supporting Information. We designed F1 scores recorded from deer or bat isolates as a positive class to construct performance metrics.

Overall, the performance of the models was feasible for the classification of genomic sequences between deer and human isolates (F1 score > 0.84) and remarkably between bat and human isolates (F1 score > 0.99) (Figure 2A and Figure S5). Among all testing classifiers, only the RR‐based model failed to classify the genomic sequences between deer and human isolates (Figure 2A and Figure S5). Confusion matrices corresponding to the individual classifiers are provided in Figure S4A (deer data set) and Figure S4B (bat data set). Bootstrapping verified the robustness of the DNN‐based model for the classification of genomic sequences possessing enriched *k*‐mers toward respective hosts irrespective of an imbalance of the sample size between hosts in each data set (Figure S6) as well as an additional “out of the bag” (OoB) data set (Figure S6, Tables S14 and S15, see Supporting Information). Altogether, these findings indicated that the enriched *k*‐mer count computed by PORT‐EK could be sufficient when predicting the likelihood of a host using multi‐genomic sequence data.

### 1.5 RaTG13 and RpYN06 are two bat coronaviruses genetically relative to deer SARS‐CoV‐2

To validate our observations that different *k*‐mers were enriched between host species, we directly compared genomic sequences isolated from US white‐tailed deer, *Odocoileus virginianus* [3], and those retrieved from the genus *Betacoronavirus* [4] across 34 bat species (Table S1). We retrieved a total number of 32,458 *k*‐mers, of which 29,660 *k‐*mers were enriched in deer and 2798 *k*‐mers enriched in bats (Figure 2B and Table S16). A clear separation of *k*‐mers enriched in deer versus those enriched in bats was observed based on the average count of individual *k*‐mers (Figure 2B). PCA illustrated a noticeable boundary of genomic sequences isolated from deer versus bats (Figure 2C). Intriguingly, based on the PCA constructed using the enriched *k*‐mer count, RaTG13 (MN996532.2) [17, 18] and RpYN06 (MZ081381.1) [17, 19] are two bat coronaviruses genetically related to deer SARS‐CoV‐2. This observation was confirmed as we also performed the phylogenetic analysis using either the complete genomic sequences (Figure 2F and Figure S7A) or the enriched *k*‐mer count (Figure 2G and Figure S7B) from deer and bats: branch points connected to RaTG13 and RpYN06 demonstrated their genomic relevance to sequences isolated from deer. Of note, we noticed the presence of two bat isolates, OM995890.1 and OM995891.1, within the cluster of isolates from deer (Figure 2C); however, as we performed literature search, these two isolates do not originate from wild bats [20], perhaps explaining our observation of the mixture of these two isolates with isolates from deer. In addition, a total of three *k*‐mers (deer, TTACAAGTTTTGGAC; bats, CTACAAGTTTTGGAC, and CGTCCGGGTGTGACC) demonstrated the highest MDI feature importance and permutation‐based importance (Figure 2D). In the final step, we applied the DNN‐based model to train the performance matrix and obtained a convincing prediction power (precision, 1; recall, 1; F1 score, 1); bootstrapping performed on 100 independent train–test splits on single genomes of isolates collected in bats born out the feasibility and effectiveness of prediction power using the enriched *k*‐mer count computed by PORT‐EK (Figure 2E).

Altogether, PORT‐EK showcased its feasibility for identifying viral genomic regions over‐represented in respective hosts, illuminating the different intrinsic tropisms of coronavirus after host domestication.

## AUTHOR CONTRIBUTIONS

**Janusz Wiśniewski**: Methodology; software; data curation; investigation; validation; formal analysis; visualization; resources; writing—original draft; writing—review and editing. **Heng‐Chang Chen**: Conceptualization; methodology; software; data curation; supervision; formal analysis; validation; investigation; funding acquisition; writing—original draft; visualization; writing—review and editing; project administration; resources.

## CONFLICT OF INTEREST STATEMENT

The authors declare no conflicts of interest.

## ETHICS STATEMENT

No animals or humans were involved in this study.

## Supporting information

Table S1. Binomial nomenclature of animal species, in which SARS‐CoV‐2 isolates retrieved for this study.
Table S2. The accession ID, geographical location, host species, and collection date of every genomic sequence of isolates collected in the US white‐tailed deer group (*Odocoileus virginianus*) (EPI_SET_240422va, https://doi.org/10.55876/gis8.240422va) in the deer dataset. Related to File S1.
Table S3. The accession ID, geographical location, host species, and collection date of every genomic sequence of isolates collected in the early 2021 human group (April 2021, EPI_SET_240422rw, https://doi.org/10.55876/gis8.240422rw) in the deer dataset. Related to File S2.
Table S4. The accession ID, geographical location, host species, and collection date of every genomic sequence of isolates collected in the late 2021 human group (November 2021, EPI_SET_240422qc, https://doi.org/10.55876/gis8.240422qc) in the deer dataset. Related to File S3.
Table S5. The accession ID, geographical location, host species, and collection date of every genomic sequence of isolates collected from 34 bat species from the NCBI Virus database in the bat dataset.
Table S6. The accession ID, geographical location, host species, and collection date of every genomic sequence of isolates collected in the human group (EPI_SET_240422qm, https://doi.org/10.55876/gis8.240422qm) in the bat dataset. Related to File S6.
Table S7. Descriptions and functionality of parameters embedded in PORT‐EK.
Table S8. Recorded running times and peak memory usage for the datasets used in this study.
Table S9. Benchmark of the functionality of PORT‐EK using the bat dataset.
Table S10. Benchmark of the functionality of PORT‐EK using the deer dataset.
Table S11. A list of enriched *k*‐mers identified in the deer dataset.
Table S12. A list of enriched *k*‐mers identified in the bat dataset.
Table S13. Details of dense neural network (DNN) architecture, hyperparameters, and training.
Table S14. The accession ID, geographical location, host species, and collection date of every genomic sequence of isolates collected in the US white‐tailed deer group (*Odocoileus virginianus*) (EPI_SET_240422oy, https://doi.org/10.55876/gis8.240422oy) in the OoB dataset. Related to File S4.
Table S15. The accession ID, geographical location, host species, and collection date of every genomic sequence of isolates collected in the human group (EPI_SET_240422xu, https://doi.org/10.55876/gis8.240422xu) in the OoB dataset. Related to File S5.
Table S16. A list of enriched *k*‐mers resulting from the comparison between genomic sequences isolated from deer and those from bats.

Figure S1. Rational design of PORT‐EK and determination of the enriched *k*‐mers.
Figure S2. Coverage landscapes of enriched *k*‐mers throughout the whole SARS‐CoV‐2 genome.
Figure S3. Landscapes of enriched *k*‐mers across the SARS‐CoV‐2 genome.
Figure S4. Prediction summary of the robustness of testing classifiers subjected to different model architectures.
Figure S5. Classification and prediction of the likelihood of SARS‐CoV‐2 host species based on the enriched *k*‐mers count.
Figure S6. Bootstrapping on the subsets of multi‐genomes in testing datasets.
Figure S7. Phylogenetic analysis of SARS‐CoV‐2 isolates from US white‐tailed deer and bats.
File S1. Deer dataset: US white‐tailed deer group (*Odocoileus virginianus*) ‐ EPI_SET_240422va, https://doi.org/10.55876/gis8.240422va. GISIAD supplemental table for deer coronavirus sequences of deer dataset.
File S2. Deer dataset: early 2021 human group (April 2021) ‐ EPI_SET_240422rw, https://doi.org/10.55876/gis8.240422rw. GISIAD supplemental table for early 2021 human coronavirus sequences of deer dataset.
File S3. Deer set: late 2021 human group (November 2021) ‐ EPI_SET_240422qc, https://doi.org/10.55876/gis8.240422qc. GISIAD supplemental table for late 2021 human coronavirus sequences of deer dataset.
File S4. OoB dataset: US white‐tailed deer group (*Odocoileus virginianus*) ‐ EPI_SET_240422oy, https://doi.org/10.55876/gis8.240422oy. GISIAD supplemental table for deer coronavirus sequences of OoB dataset.
File S5. OoB dataset: human group ‐ EPI_SET_240422xu, https://doi.org/10.55876/gis8.240422xu. GISIAD supplemental table for human coronavirus sequences of OoB dataset.
File S6. Bat dataset, human group ‐ EPI_SET_240422qm, https://doi.org/10.55876/gis8.240422qm. GISIAD supplemental table for human coronavirus sequences of bat dataset.

## Data Availability

The data that supports the findings of this study are available in the supplementary material of this article. All data generated by PORT‐EK are available in the main text and Supporting Information. Readouts from PORT‐EK, including a list of enriched *k*‐mers identified in datasets, and enriched *k*‐mers retrieved from the comparison of isolates between deer and bats, recorded running times and peak memory usage for the datasets used in this study, and details of dense neural network (DNN) architecture, hyperparameters, and training are provided in Supporting Tables, respectively and available for public download. We documented the complete PORT‐EK pipeline and analytical scripts in GitHub, which is available for public download at https://github.com/wis-janusz/PORT-EK. The rest of the analytical scripts are available for public download at https://github.com/HCAngelC/PORT-EK-Kmer-analysis. Code and raw data related to Figures 1 and 2 are archived on GitHub (https://github.com/HCAngelC/PORT-EK-Kmer-analysis). Supplementary materials (methods, figures, tables, files, graphical abstract, slides, videos, Chinese translated version, and update materials) may be found in the online DOI or iMetaOmics http://www.imeta.science/imetaomics/.
