# Supplementary material for: Identification of potential SARS‐CoV‐2 genomic regions representing hallmarks for adaptation to different hosts
Source: IMetaOmics. 2025 May 2;2(2):e70019. doi: 10.1002/imo2.70019 (PMC12806211; doi:10.1002/imo2.70019)
Supplement: Supplementary file 2 — Figure S1. Rational design of PORT‐EK and determination of the enriched k‐mers. Figure S2. Coverage landscapes of enriched k‐mers throughout the whole SARS‐CoV‐2 genome. Figure S3. Landscapes of enriched k‐mers across the SARS‐CoV‐2 genome. Figure S4. Prediction summary of the robustness of testing classifiers subjected to different model architectures. Figure S5. Classification and prediction of the likelihood of SARS‐CoV‐2 host species based on the enriched k‐mers count. Figure S6. Bootstrapping on the subsets of multi‐genomes in testing datasets. Figure S7. Phylogenetic analysis of SARS‐CoV‐2 isolates from US white‐tailed deer and bats. File S1. Deer dataset: US white‐tailed deer group (Odocoileus virginianus) ‐ EPI_SET_240422va, https://doi.org/10.55876/gis8.240422va. GISIAD supplemental table for deer coronavirus sequences of deer dataset. File S2. Deer dataset: early 2021 human group (April 2021) ‐ EPI_SET_240422rw, https://doi.org/10.55876/gis8.240422rw. GISIAD supplemental table for early 2021 human coronavirus sequences of deer dataset. File S3. Deer set: late 2021 human group (November 2021) ‐ EPI_SET_240422qc, https://doi.org/10.55876/gis8.240422qc. GISIAD supplemental table for late 2021 human coronavirus sequences of deer dataset. File S4. OoB dataset: US white‐tailed deer group (Odocoileus virginianus) ‐ EPI_SET_240422oy, https://doi.org/10.55876/gis8.240422oy. GISIAD supplemental table for deer coronavirus sequences of OoB dataset. File S5. OoB dataset: human group ‐ EPI_SET_240422xu, https://doi.org/10.55876/gis8.240422xu. GISIAD supplemental table for human coronavirus sequences of OoB dataset. File S6. Bat dataset, human group ‐ EPI_SET_240422qm, https://doi.org/10.55876/gis8.240422qm. GISIAD supplemental table for human coronavirus sequences of bat dataset. [file IMO2-2-e70019-s002.docx]

Supporting Information for

**Identification of potential SARS-CoV-2 genomic regions representing hallmarks for adaptation to different hosts**

**Running title**: **PORT-EK captures enriched *k*-mers between different SARS-CoV-2 hosts**

Janusz Wiśniewski^1^ and Heng-Chang Chen^1,2,*^

^1^Quantitative Virology Research Group, Population Diagnostics Center, Łukasiewicz Research Network – PORT Polish Center for Technology Development, Stablowicka 147, 54-066 Wrocław, Poland

^2^The Laboratory of Quantitative Virology, Centre for Advanced Materials and Technologies, Warsaw University of Technology, 19 Poleczki St, 02-822, Warsaw, Poland

^*^Correspondence: [heng-chang.chen@port.lukasiewicz.gov.pl](mailto:heng-chang.chen@port.lukasiewicz.gov.pl) (Heng-Chang Chen)

**This PDF file includes:**

Supporting Text

Supporting Figures S1 to S7

Supporting Files S1 to S6

**Table of Contents**

Introduction…………………………………………………………………………………………………1

**METHODS……………………………………………………………………………………………….. 5**

Source of Data………………………………………………………………………………………………5

Calculation of the coverage of enriched *k*-mers over the SARS-CoV-2 genome…………………………..6

Calculation of the quantitative enrichment of enriched *k*-mers at a genic level……………………………6

Data visualization………………………………………………………………………………………….. 7

Statistics…………………………………………………………………………………………………….8

**COMPUTATIONAL METHODS……………………………………………………………………….. 8**

Model overview, requirements, and parameters ……………………………………………………………8

Step breakdown of PORT-EK………………………………………………………………………………9

*K*-mer extraction, count matrix, and descriptive statistics used in PORT-EK…………………………….. 9

Determination of the optimal *k* value …………………………………………………………………….11

Over-represented and enriched *k*-mer identification ………………………………………………………11

Enriched *k-*mers mapping …………………………………………………………………………………12

Prediction of the likelihood of the host……………………………………………………………………12

Evaluation of the DNN-based classifier using bootstrapping……………………………………….13

**SUPPORTING FIGURES……………………………………………………………………………….14**

Figure S1…………………………………………………………………………………………………..14

Figure S2…………………………………………………………………………………………………..15

Figure S3…………………………………………………………………………………………………..16

Figure S4…………………………………………………………………………………………………..19

Figure S5…………………………………………………………………………………………………..20

Figure S6…………………………………………………………………………………………………..21

Figure S7…………………………………………………………………………………………………..22

**SUPPORTING FILES………………………………………………………………………………….. 25**

File S1……………………………………………………………………………………………………..25

File S2……………………………………………………………………………………………………..26

File S3……………………………………………………………………………………………………..27

File S4……………………………………………………………………………………………………..28

File S5…………………………………………………………………………………………………..…29

File S6……………………………………………………………………………………………………..30

**REFERENCES………………………………………………………………………………………….. 31**

**Introduction**

Since the beginning of the SARS-CoV-2 pandemic, the demands for artificial intelligence (AI) and machine learning methodologies have rapidly elevated to efficiently digest an abundant number of SARS-CoV-2 variants’ sequences that are generated on a daily basis. Nowadays, language models, e.g. natural language processing (NLP) algorithms and the *k*-mer-based approach have been widely applied to mine biological information masked under every sequence. In the former approach, language models provide the opportunity to process and understand text-based sequencing data in a more human-like manner. Liu et al. utilized the principle of NLP to learn the information of SARS-CoV-2 gene sequences and used them to construct the model represented by graph attention networks, an RNA virus transmission network, to recapitulate the topological character of COVID-19 transmission [1]. Singh et al. utilized fundamental algorithms in NLP to reveal the characteristics and evolution of SARS-CoV-2 genomes [2]. Bist et al. developed a computational model that recognizes significant mutational sequences in SARS-CoV-2 spike protein sequences based on escape feature identification using NLP [3]. Tong et al. applied NLP to develop ViMRT, a text-mining tool to search engine for automated virus mutation recognition [4]. Zhou et al. developed an NLP-based transformer-based mutation prediction framework, namely TEMPO to predict the mutations alongside SARS-CoV-2 evolution [5].

Methodologies using *k-*mers representation directly compare the counts of nucleotide sequences of the length *k* between samples [6,7]. A common step is to break a reference sequence into *k*-mers and use them to create a hash table. In parallel, target sequences are broken into *k*-mers and queried against the hash table to check for shared *k*-mers [8–10]. Different *k*-mer-based models have been developed to optimize sequence analysis and comparison, such as the *k*-mer sparse matrix model for sequence comparison [11] and an anomaly detection algorithm [12–15]. The former method denotes the types and sites of *k*-mers in genetic sequence based on sequence comparison [11], whereas the latter method tends to identify outliers in a dataset [12–15]. These approaches have gained momentum in high-throughput sequencing data analysis [16–20] and have been central to the field of metagenomics, where they are used to discover unique *k*-mer signatures to classify organisms [21] and capture biological variations and functional annotation in RNA-seq data [6,22]. More recently, the *k*-mer counting strategy has also been applied to identify genomic sequence signatures harboring mutations across thousands of SARS-CoV-2 genomic sequences [23]. Here we present a *k*-mer-based approach, namely the Pathogen Origin Recognition Tool using Enriched *K*-mers (PORT-EK), which allows for a comparison of different multi-genomic datasets encompassing viral genomic sequences captured from two host species and the identification of over-represented genomic regions represented by *k*-mers correlated to specific hosts.

It is important to stress that, using PORT-EK, we sought any viral genomic region that is quantitatively enriched in one host. Thus, we calculated the difference in the average count of individual *k-*mers between two host species (deer versus humans or bats versus humans) within the same dataset and tested their statistical significance designated as its *p*-value (detailed in **Supporting Information**) in the deer (**Figures 1A**-**1C**) and bat datasets (**Figure 1D**). Of note, in this work, the positive value denotes the average count of each *k*-mer enriched from animal hosts (deer and bats); whereas the negative value denotes the enrichment related to humans. For *k*-mers over-represented in deer isolates, we focused on the ones that were over-represented against both the early and late 2021 human groups to achieve a greater probability that the identified *k*-mers result from a consecutive period of intrahost selection pressure (**Figure 1C**). We cannot perform this selection on the *k*-mers retrieved in the bat dataset because no clear temporal boundary exists in relation to bat isolates restricted in the same geographical regions nor in human isolates used in the bat dataset. In this circumstance, we analyzed a total of 263 available betacoronavirus sequences isolated from bats (mostly from China) from 2005 to 2022 against 2081 sequences from human isolates collected from the early pandemic in this study (see **METHODS**, **Source of Data** below).

**Classification and prediction**

In this work, the proposed model used for the classification and prediction of the likelihood of host species was constructed using the total count of enriched *k*-mers. To reduce data and model dimensions and improve performance, *k*-mers with highly linearly correlated counts were grouped, and a single *k*-mer from each group was selected. This reduced the number of *k*-mers identified in deer and bat isolates from 1,470 to 211 and from 32,917 to 489, respectively. We performed PCA to reveal the discrepancy between the genomic sequences of isolates present in different hosts based on the enriched *k*-mer count (**Figures S5A** and **S5B**). Of note, we used PCA for the visualization of the distribution of the *k*-mer count; classification itself was performed based on the enriched *k*-mer count. The genomic sequences from bat isolates were linearly separable from those from human isolates (**Figure S5B**), whereas sequences from deer isolates were not (**Figure S5A**), implying that the diversity of SARS-CoV-2 genomic sequences between bat and human isolates was wider than that between deer and human isolates.

We further utilized the random forest model to estimate the relative importance of *k*-mers and achieved F1 scores equal to 0.83 and 1 for the classification of *k*-mers over-represented in deer and bat isolates versus human isolates, respectively. The mean decrease in impurity (MDI) feature importance can be biased [24,25]; thus, we additionally calculated permutation-based importance [26] to reinforce the accuracy of the classification. Although most enriched *k*-mers in deer and human isolates cluster together at low importance values, a total of seven enriched *k*-mers demonstrated the highest significance of both importance metrics (**Figure S5C**). Even though the classifier failed to compute permutation-based importance in the bat dataset (**Figure S5D**), we were able to detect a total of six distinguishable enriched *k*-mers against all inputs based on MDI importance (**Figure S5D**).

To ensure the robustness of our classifier and further estimate whether or not our models were also applicable to other genomic data, we retrieved additional SARS-CoV-2 genomic sequences from deer (n = 70, between October and December 2021) and human (n = 6,705, January 2022) isolates within a different period of time from the multi-genomic data that were previously examined in this study (detailed in **METHODS**, **Source of Data** below). We named this set of genomic data out of the bag (OoB) and used them to evaluate the effectiveness and feasibility of using our established models. All models, except for RR, offered acceptable performance, with DNN being the most competent (F1 score equal to 0.66) (**Figure S5E**). The confusion matrices generated from the classifier built up using the OoB dataset are provided in **Figure S4C**.

Given that the sample size of input sequences varies in each dataset, we performed bootstrapping to generate 100 independent train–test splits and retained the DNN-based model to examine whether the varying sample sizes biased the accuracy of our models. We plotted the distribution of the F1 scores for each dataset (the deer and OoB datasets, **Figure S6A**; the bat dataset, **Figure S6B**). We observed the robustness of the DNN-based model for the classification of genomic sequences possessing enriched *k*-mers toward respective hosts, indicating that the DNN-based model could serve as a default choice for prediction while using PORT-EK. Details of DNN architecture is provided in **Table S13**.

In this work, we also applied the phylogenetic analysis to either the complete genomic sequences (**Figures 2F** and **S7A**) or the enriched *k*-mer count (**Figures 2G** and **S7B**) to confirm the robustness of the enriched *k*-mer count as a representative of the complete genomic sequences. It is, however, important to note that the quality and diversity of the input sequences may limit the resolution of the trees. One potential issue with the phylogenetic tree-based sampling method can be that there is no consensus about the correct phylogenetic trees for a given set of taxa; another is that information of a set of taxa cannot be relied only on the measurement of the taxa [27]. A continuation of the improvement of the taxonomic classification of viruses will be requisite to elevate the accuracy of the phylogenetic tree-based sampling method.

**Limitations of this study**

One of the limitations is that we cannot identify unique enriched *k*-mers present in different species. One of the reasons may result from the similarity of genomic sequences across isolates retrieved in this study. Despite this limitation, we were able to seek the significant enrichment of viral genomic regions (namely *k*-mers in this study) and utilized the enriched *k*-mer count for classification and prediction. Second, we cannot ascertain whether enriched *k*-mers identified using PORT-EK cover all dominant genomic regions resulting from host domestication. Undiscovered *k*-mers may be due to sequences of isolates that are either not yet characterized or not included in the present dataset. Of note, we only chose complete genomic sequences for analyses in this study. This issue is also referred to as the sample size in some species, e.g. the genomic sequences in deer isolates, is small in this study. We bootstrapped human isolates into several subsets with the equal size to animal isolates and were capable of covering more than 80% of enriched *k*-mers compared to those retrieved from upsampling input sequences, suggesting that enriched *k*-mers retrieved from total input sequencing using PORT-EK remain robust. Furthermore, in this study, we did not investigate and compare enriched *k*-mers identified from multiple periods, rendering it difficult to track their evolutionary trajectories in circulation within a host. Indeed, based on PORT-EK, we identified enriched *k*-mers with a statistical test that assumes independence; the frequencies of the *k*-mers are however not independent in the real world: viruses evolve biologically so a mutation that appears early will remain in all the descendants until it mutates again. Further advanced statistical methods, e.g. phylogenetic regression will be required to perform statistics on samples of biological sequences. Finally, at present, the number of publicly available SARS-CoV-2 genome sequences in many host species remains limited, rendering the calculation of the statistical significance of enriched *k*-mers difficult. PORT-EK has the potential to unveil enriched *k*-mers across a wider range of host species when more SARS-CoV-2 genomes are sequenced.

Technically, in the PORT-EK pipeline, our filtering strategy for the selection of enriched *k*-mers is based on the frequency of *k*-mers, the difference in the average counts of *k*-mers between two species, and RMSE, and other filtering methods, such as minimizers and convolutional neural networks (CNNs), will also be implemented to reinforce the accuracy of PORT-EK. In addition, it is important to stress that when evaluating the robustness of PORT-EK, we noticed that some enriched *k*-mars cannot be aligned to the reference genome. We assume that it may be due to (1) the choice of the mapping algorithm or (2) the fact that the reference genome chosen cannot cover all SARS-CoV-2 variants. In the future, the selection of multiple reference genomes and mapping algorithms should also be considered to reinforce the completeness of the final readouts. Finally, we propose that the usage of PORT-EK should be coupled with other genomic approaches, such as RNA-seq, and single-cell RNA-seq, to augment the ramifications of the identified *k*-mers.

**METHODS**

**Source of Data**

In this study, based on retrieved multi-genomic sequences, we prepared three datasets, namely the deer, bat, and OoB datasets. Each dataset consists of genomic sequences of SARS-CoV-2 isolates from two different host species, allowing the comparison of the enrichment of *k*-mers between each other. Of note, only complete genome sequences were included in this study.

In the deer dataset, 336 genomic sequences of SARS-CoV-2 variants collected from North America white-tailed deer, *Odocoileus virginianus* [28], between October and December 2021 were utilized (**File S1** and **Table S2**). We compared sequences isolated from deer with the sequences from human isolates (in USA) collected at two different collection periods: 21,906 sequences collected in April 2021 (namely the early 2021 human group in the main text, **File S2** and **Table S3**) and 11,525 sequences collected in November 2021 (namely the late 2021 human group, **File S3** and **Table S4**).

With respect to the OoB dataset, 70 additional SARS-CoV-2 sequences collected between October and December 2021 in US white-tailed deer, *Odocoileus virginianus* [28], were used (**File S4** and **Table S14**) and 6,705 sequences from human isolates collected in January 2022 were used (**File S5** and **Table S15**).

Of note, we used this dataset to evaluate the effectiveness and feasibility of our established prediction models that will be detailed in the following.

In the bat dataset, we retrieved a total of 263 available betacoronavirus sequences isolated from 34 bat species (**Table S1**) in the NCBI Virus database (**Table S5**). Sequences were collected mainly from China from 2005 to 2022. 2081 sequences from human isolates were collected also from various geographies between December 2019 and February 2020 (**File S6** and **Table S6**) for analyses.

In summary, the sequences used in this study are available from either the GISAID EpiCoV or NCBI Virus database as follows:

1. Deer dataset: US white-tailed deer group (*Odocoileus virginianus*) - EPI_SET_240422va,<https://doi.org/10.55876/gis8.240422va> (**File S1** and **Table S2**).

2. Deer dataset: early 2021 human group (April 2021) - EPI_SET_240422rw,<https://doi.org/10.55876/gis8.240422rw> (**File S2** and **Table S3**).

3. Deer dataset: late 2021 human group (November 2021) - EPI_SET_240422qc,<https://doi.org/10.55876/gis8.240422qc> (**File S3** and **Table S4**).

4. OoB dataset: US white-tailed deer group (*Odocoileus virginianus*) - EPI_SET_240422oy,<https://doi.org/10.55876/gis8.240422oy> (**File S4** and **Table S14**).

5. OoB dataset: human group - EPI_SET_240422xu,<https://doi.org/10.55876/gis8.240422xu> (**File S5** and **Table S15**).

6. Bat dataset: bat group - NCBI Virus accession numbers (**Table S5**).

7. Bat dataset: human group - EPI_SET_240422qm,<https://doi.org/10.55876/gis8.240422qm> (**File S6** and **Table S6**).

Of note, the supplementary files for GISAID data are provided in the pdf format and used as is without any modification.

**Calculation of the coverage of enriched *k*-mers over the SARS-CoV-2 genome**

Once the single-nucleotide positions overlaid by enriched *k*-mers were characterized, we calculated the *k*-mers coverage of each genomic locus based on the total number of enriched *k*-mers overlaid. For every locus, based on the competition of the sum of enriched *k*-mers, separated by corresponding hosts, we categorized the tropism of each locus into three groups: exclusive, favorable, and comparable sites (**Figures 1G**-**1J**). Exclusive sites were referred to as the scenario that all *k*-mers are enriched in the same host; favorable sites were referred to as the scenario that the total number of enriched *k*-mers were superior in one host species than another within the same dataset; comparable sites were referred to as the total number of enriched k-mers were equal between two host species within the same dataset.

**Calculation of the quantitative enrichment of enriched *k*-mers at a genic level**

The enrichment of enriched *k-*mers exclusively enriched in animal or human isolates at individual SARS-CoV-2 genes was calculated by Equation (2).

$E_{exclusive}(k,i)=(\sum_{k\in G} k/\sum_{i\in G} i)/S$ (2)

where $E_{exclusive}$ is the enrichment of a total number of enriched *k-*mers $k$ covering given SARS-CoV-2 viral genomic loci $i$ normalized by the size $S$ of a given SARS-CoV-2 gene $G$.

The enrichment of enriched *k-*mers that were present in both host species and more quantitatively dominant in either animal or human isolates at individual SARS-CoV-2 genes was calculated by Equation (3).

$E_{favorable}(k_{a,}i_{a},k_{b},i_{b})=(((\sum_{k_{a}\in G} k_{a}/\sum_{i_{a}\in G} i_{a})-(\sum_{k_{b}\in G} k_{b}/\sum_{i_{b}\in G} i_{b}))/S)$ (3)

where $E_{favorable}$ is the enrichment of a total number of enriched *k*-mers $k_{a}$, which cover given SARS-CoV-2 viral genomic loci $i_{a}$ in one species that dominate a total number of the enriched 15 nt *k*-mers $k_{b}$, which cover given SARS-CoV-2 viral genomic loci $i_{b}$ in another species followed by a normalization by the size $S$ of a given SARS-CoV-2 gene $G$. Of note, given that the total number of *k*-mers retrieved in the comparable group was identical in both species, this group was thus excluded from this analysis. The calculation of the enrichment is visualized in **Figures S3A**. The genomic positions of SARS-CoV-2 protein–coding genes, including the gene encoding NSP1 (266 bp - 805 bp), the gene encoding NSP2 (806 bp - 2719 bp), the gene encoding NSP3 (2720 bp - 8554 bp), the gene encoding NSP4 (8555 bp - 10054 bp), the gene encoding NSP5 (10055 bp - 10972 bp), the gene encoding NSP6 (10973 bp - 11842 bp), the gene encoding NSP7 (11843 bp - 12091 bp), the gene encoding NSP8 (12092 bp - 12685 bp), the gene encoding NSP9 (12686 bp - 13024 bp), the gene encoding NSP10 (13025 bp - 13441 bp), the gene encoding NSP11 (13442 bp - 13480 bp), the gene encoding NSP12 (13481 bp - 16236 bp), the gene encoding NSP13 (16237 bp - 18039 bp), the gene encoding NSP14 (18040 bp - 19620 bp), the gene encoding NSP15 (19621 bp - 20658 bp), the gene encoding NSP16 (20659 bp - 21552 bp), the gene encoding the S protein (21563 bp - 25384 bp), the *orf3a* gene (25393 bp - 26220 bp), the gene encoding the E protein (26245 bp - 26472 bp), the gene encoding the M protein (26523 bp - 27191 bp), the *orf6* gene (27202 bp - 27387 bp), the *orf7*a gene (27394 bp - 27759 bp), the *orf7b* gene (27756 bp - 27887 bp), the *orf8* gene (27894 bp - 28259 bp), the gene encoding the N protein (28274 bp - 29533 bp), the *orf10* gene (29558 bp - 29674 bp), were recorded from NCBI Reference Sequence: NC_045512.2 - Severe acute respiratory syndrome coronavirus 2 isolate Wuhan-Hu-1, complete genome ([https://genome.ucsc.edu/cgi-bin/hgTracks?db=wuhCor1&lastVirtModeType=default&lastVirtModeExtraState=&virtModeType=default&virtMode=0&nonVirtPosition=&position=NC_045512v2%3A1%2D29903&hgsid=2236086162_7owbHRSgRbADlT8C9GSdZ4z6bwp5](https://genome.ucsc.edu/cgi-bin/hgTracks?db=wuhCor1&lastVirtModeType=default&lastVirtModeExtraState=&virtModeType=default&virtMode=0&nonVirtPosition=&position=NC_045512v2:1-29903&hgsid=2236086162_7owbHRSgRbADlT8C9GSdZ4z6bwp5)). 100 (**Figure S3B**) and 500 (**Figure S3C**) enriched *k*-mers in the deer dataset and 1,000 (**Figure S3D**) and 5,000 (**Figure S3E**) enriched *k*-mers in the bat dataset were bootstrapped with replacement and this process was repeated 5,000 times in order to confirm analyses performed using the total number of enriched *k*-mers. The analytical scripts are available for public download at<https://github.com/HCAngelC/PORT-EK-Kmer-analysis>.

**Data visualization**

***Volcano plot representation of over-represented k-mers***

Volcano plots tailored for the representation of over-represented *k*-mers were created using Python 3.11. In this plot, the x-axis represents the average *k*-mers count coverage and the y-axis represents -log_10_(*p*-value). An additional scatter plot (**Figure 1C**) transformed from the volcano plots in the deer dataset was generated to highlight *k*-mers over-represented in deer isolates in contrast to isolates from both early and late 2021 human groups.

***Line plot representation of SARS-CoV-2 genomic loci overlaid with enriched k-mers***

The line plots representing the landscape of the SARS-CoV-2 genomic sequence per locus, in which nucleotides overlay with enriched *k*-mers (**Figures 1I** and **1J**) were created using the default R build-in function geom_segment().

***Phylogenetic analysis***

Phylogenetic trees were constructed based on either multiple sequence alignment (MSA-based trees, **Figures 2F** and **S7A**) or enriched *k*-mer counts (*k*-mer-based trees, **Figures 2G** and **S7B**). Multiple sequence alignments were created using Kaling 3.4.0 [29]. For MSA-based trees, pairwise distances were calculated from alignments using DistanceCalculator class from Biopython’s TreeConstruction module [30]. For *k*-mer-based trees, pairwise distances were calculated form enriched *k*-mer count matrices generated by PORT-EK using pdist function from scipy [31]. In both cases, the distances were used to construct trees using Neighbour Joining method [32] with Biopython’s TreeConstruction module [30]. MSA-based trees were rooted with the reference sequence, whereas *k*-mer-based trees were unrooted. All trees were saved in PhyloXML format (available at Github <https://github.com/wis-janusz/PORT-EK>).

**Statistics**

All statistical tests were performed with Python and R (version 4.3.3) with default options. Details are provided where appropriate in the main text, figure legends and **Supporting Information**.

**COMPUTATIONAL METHODS**

**Model overview, requirements, and parameters**

PORT-EK is a pipeline that compares sequences from two different multi-genomic datasets and identifies over-represented *k-*mers in respective datasets. In this study, we further utilize the count of enriched *k-*mers as the predictor variable to classify the likelihood of respective hosts (detailed in the following section **Prediction of the likelihood of the host**), in which *k-*mers are over-represented. This pipeline is written in Python 3.11 (<https://github.com/wis-janusz/PORT-EK>), using: biopython 1.81, numpy 1.26.2, pandas 2.1.4, seaborn 0.13.2, matplotlib 3.8.2, networkx 3.2.1, scipy 1.11.4, keras 2.15.0 and scikit-learn 1.3.2. It also requires the support of Jupyter notebooks to execute several critical steps involved in PORT-EK, including *k*-mer filtering, statistics, and identification of enriched *k*-mers coupled with mutations.

While using PORT-EK, input files should be in the fasta format with headers either in GISAID or NCBI Virus formats. All sequences in one input file correspond to the same host; multiple input files can be pooled for the same host if required. PORT-EK allows the comparison of two independent sources of multi-genomes. In this study, each dataset consists of multi-genomes isolated from two different host species. Of note, a set of tunable parameters includes the *k-*mer length *k*, conservation threshold *c*, allowed rare *k*-mer mismatches *m*, minimum root mean square error $min_{RMSE}$, allowed mapping mismatches $m_{map}$ , and allowed mapping offset $l_{map}$ are recommended to be adjusted whenever a new genomic dataset is applied. Description and the functionality of each parameters were provided in **Table S7**. The details of the parameters and their settings are discussed later in the following section. Recorded running times and peak memory usage for the data sets used in this study are presented in **Table S8**.

**Step breakdown of PORT-EK**

The overview of each step in PORT-EK is summarized as follows: First, we extracted all *k*-mers of length *k* from multi-genomic datasets and recorded their counts. At this stage, we discarded the *k*-mers with any ambiguous nucleotides (first layer of filtering) and constructed a count matrix containing counts of all non-redundant *k*-mers from all input multi-genomic sequences. Second, we calculated the frequency of appearance of *k*-mers (detailed in the following content) and the average counts of all filtered *k*-mers present in the same host isolates. We kept *k*-mers that passed through a tunable frequency threshold and marked them as “common” *k*-mers; for those that failed, we marked them as “rare” *k*-mers (**Figure S1B**; the second layer of filtering). Third, we computed the difference in the average count of *k*-mers and root mean square error (RMSE, detailed in the following content) between the host isolates (third layer of filtering). Of note, it was the step at which the optimal length of *k*-mers was determined. The rationale and the calculation of the specificity and efficiency of *k*-mers for the determination of the optimal length of *k*-mers are detailed in the following content. From a pool of the common *k*-mers, we retrieved those with statistical significance, showing differences in the average count between two host isolates within the same dataset. They were the *k*-mers over-represented in the isolates from the same host (**Figure S1B**). In parallel, we inspected the sequence of rare *k*-mers and pooled them with common *k*-mers when sequences of rare *k*-mers resembled any common *k*-mer. Finally, to obtain the final output of the *k*-mers, we once again applied an RMSE-based filter to those *k*-mers over-represented in isolates from the same host, enabling the concentration of enriched *k*-mers identified in one specific host (the fourth layer of filtering). The hierarchical filtering procedure used for the identification of enriched *k*-mers is illustrated in **Figure S1B**.

***K*-mer extraction, count matrix, and descriptive statistics used in PORT-EK**

We first extracted *k*-mers of the length *k* with overlapping sequences using a sliding window moving every nucleotide and placing them in the same hash tables. *K*-mer sequences and the first nucleotide position were used as indices for constructing this position matrix. A single ID was assigned to each hash tables. Host species were denoted to corresponding hash tables of *k*-mers.

Next, the indices were read to construct a matrix containing the count of every *k*-mer in every hash table. Inside a matrix, rows indicate the *k*-mer sequence, and columns indicate the hash tables. *K*-mers with a poly(A) sequence were discarded. We further measured the appearance of *k*-mers, representing the “frequency” for each host. Frequency was calculated based on the ratio between the total number of *k*-mers in one hash table and the total number of hash tables in the host. This is the second filter, namely the rarity filter in PORT-EK (**Figure S1B**). Based on the assumption that *k*-mers containing meaningful information should not be very rare within the hosts [23], only *k*-mers that are at least *c* % conserved to the respective host were retained for further analyses. Lower settings of *c* allow the pipeline to capture rarer variants that may still be meaningful at the cost of increased computation time, memory usage, and difficulty of interpretation; the opposite is true for higher settings. We found that *c* of 1% is sufficient for the deer and OoB datasets, whereas *c* of 50% is required for the bat dataset. We assumed that it is due to the fact that in this work different bat species were included, whilst genomic sequences in deer were probably more closely related. We termed the *k-*mers that pass the rarity filter “common *k*-mers”, whereas those that do not were named “rare *k*-mers”.

We further computed the following three statistics on common *k*-mers:

(1) the average count of each *k*-mer for two individual hosts, $n_{nh}$ and $n_{hi}$, where $n_{hi}$ indicates the identifier of a particular human host group.

(2) the difference in the average count of each *k*-mer between two hosts: $\Delta n_{i}=n_{nh}-n_{hi}$, and

(3) the root mean square error (RMSE) of said changes:

$RMSE=\sqrt{\frac{1}{k}\sum_{i=1}^{k} {(\Delta n_{i})}^{2}}$ (1)

where $k$ is the total number of human groups.

The difference in the average count of each *k*-mer was used throughout the whole pipeline as metrics of enrichment. In this work, the positive value denotes the average count of each *k*-mer enriched from animal hosts (deer and bats); whereas, the negative value denotes the enrichment related to humans. RMSE was used for an unsigned measure of the degree of enrichment. In other words, the larger the RMSE is, the more pronounced the difference between two comparing hosts is. Finally, the statistical significance of $\Delta n_{i}$ was tested using Fisher’s exact test. The differences with *p*-values less than 0.01 were deemed significant.

**Determination of the optimal *k* value**

We selected the optimal *k* value based on the percentage of non-repeating *k*-mers among the common *k*-mers. To only measure significant changes, we rounded the percentage to the whole percent. The optimal *k* value was defined to be the lowest *k* at which the percentage of non-repeating *k*-mers stopped increasing. We compared the percentage of non-repeating *k*-mers in common *k*-mers of length *k* from the whole data set (**Figure S1C** and **S1D**). In this study, we compared $k$ values of 5, 7, 9, 11, 13, 15, 17, and 19. We observed that the percentage of unique *k*-mers shows an increase while the value $k$ increases. A plateau appears when the $k$ value is bigger than 13. Additionally, we used the size of the *k*-mer count matrix in kilobytes (kB) as a measurement of computational efficiency (**Figures S1E** and **S1F**).

**Over-represented and enriched *k*-mer identification**

Over-represented *k*-mers were retrieved from a pool of common *k*-mers (**Figure S1B**) based on three mentioned statistical strategies (the third layer of filtering). Only *k*-mers with *p*-values less than 0.01 were retained. The parameter $\Delta n_{i}$ was used to determine in which species *k*-mers were enriched. In this study, a positive value of $\Delta n_{i}$ indicated that *k*-mers were enriched in isolates from deer or bats, whereas a negative value indicated *k*-mers were enriched in human isolates. It is important to note that, with respect to *k*-mers over-represented in deer isolates, we only retained the ones that were over-represented against both early and late human 2021 groups. *K*-mers with *p*-values greater than 0.01 were discarded.

At this step, we re-examined the sequence similarity between rare and over-represented *k*-mers (**Figure S1B**). Rare *k-*mers with the superior sequence similarity (two-nucleotides mismatches, the parameter *m*, allowed) to any of common *k*-mers were added to a pool of over-represented *k*-mers. This step is however optional in the PORT-EK pipeline. We constructed a graphic network, in which nodes represent a pool of the over-represented *k*-mers and rejoining rare *k*-mers, and edges represent the similarity between every pair of an over-represented *k*-mer and a rejoining rare *k*-mer, allowing the visualization of the distribution of both subsets of the *k*-mers.

At the final step of the PORT-EK pipeline, we applied, once again, the statistical tools implemented in the third layer of filtering to those rejoining rare *k*-mers. We passed all over-represented and rejoining rare *k*-mers to the fourth RMSE filter in order to retrieve enriched *k*-mers as a final output (**Figure S1B**). *K-*mers that have an RMSE less than $min_{RMSE}$ were discarded. Of note, the parameter $min_{RMSE}$ is adjustable in the range between 0 and 1, allowing fine-tuning of the sensitivity of PORT-EK. As the value $min_{RMSE}$ is approaching 1, the strength of the fourth filter is stricter. In this study, the default setting $min_{RMSE}$ was equal to 0.1. The rest of the *k*-mers were considered to manifest significant enrichment and were designated to corresponding hosts. The enriched *k*-mer matrix was then transposed, as IDs were listed in rows and *k*-mers in columns. Host species were assigned to enriched *k*-mers shown in columns. Column labeling the host of the viral samples was added. Host species labels were numerical and binary: deer or bats were labeled as 1; humans as 0.

**Enriched *k-*mers mapping**

In this study, we established a tailored algorithm using Python regular expression library, RegEx, enabling the mapping of the enriched *k*-mers throughout the reference genome, severe acute respiratory syndrome coronavirus 2 isolate Wuhan-Hu-1, complete genome NC_045512.2, curated in the National Center for Biotechnology Information (NCBI) database (<https://www.ncbi.nlm.nih.gov/nuccore/1798174254>). Only *k*-mers that were present one time in individual viral genomic sequences were used for mapping to minimize the probability of the presence of ambiguous *k*-mers. The genomic position corresponding to the first nucleotide from each selected *k-*mer was used as *k*-mer indices to form a matrix. In principle, 15 nt substrings in sliding windows consecutively shifting per nucleotide were read through over the whole reference genome. Every substring was compared with mentioned *k*-mer sequences with up to *i* mismatches, with *i* increasing from 0 to $m_{map}$, the maximum number of mismatches.

To align enriched *k*-mers throughout the SARS-CoV-2 genome per site, we sought the lowest number of mismatches followed by the lowest starting position difference. If no substrings were found with less than $m_{map}$ mismatches or less than mapping offset $l_{map}$, the starting position difference, and no match were returned. By default, $m_{map}$, it is set to the same number as *m* denoted as the number of mismatches, at the step of the identification of rare *k*-mers, and $l_{map}$ is set to 1000 (both parameters are tunable). In this study, we set $m_{map}$ equal to 2 and $l_{map}$ equal to 1000 as default. Based on the collection of substrings, we could depict the viral genomic positions overlaid with enriched *k*-mers at a single-nucleotide level.

**Prediction of the likelihood of the host**

We utilized the counts of enriched *k*-mers as the predictor variable for the prediction of the likelihood of the host. Here we combined the genomic sequences of isolates from the early and late 2021 human groups to simplify the procedure of classification and prediction. In principle, we first calculated a correlation matrix of *k*-mer using pandas [33] corr() function and constructed a graph, in which nodes representing each enriched *k*-mer and edges connecting nodes representing *k*-mers with a correlation coefficient of more than 0.9 (including loops). The connected components shown in this graph represent the groups of *k*-mers with a high correlation of enriched *k*-mer counts. Based on enriched *k*-mer counts, we were able to calculate the absolute value of the correlation coefficient for each *k*-mer associated with the respective host and set it as a node attribute *y_corr*. Finally, *k*-mers (illustrated as nodes in the graph) coupled with the largest *y_corr* from individual connected components in a graph were selected as the so-called linearly independent *k*-mer set.

The linearly independent *k*-mer set was split into training and test sets at a ratio of 0.7 to 0.3, with stratification associated with the respective host. The importance of particular *k*-mers for classification was calculated based on a mean decrease in impurity importance (MDI) and permutation importance [34] using scikit-learn’s RandomForestClassifier with balanced class_weight; remaining hyperparameters at default values. F1 score obtained from linearly independent *k*-mers enriched in deer and bats were set as positive values, serving as a metric. *K*-mers with the values of importance equal to zero or negative values of importance were removed from the dataset, and were, once again, split into training and test sets at the same mentioned ratio. Of note, given such a prevalent imbalance of the sample size of genomic sequences retrieved from animal species versus humans [35], rendering the measure of ROC AUC (Area under the ROC Curve) uninformative, in this study, we thus recorded F1 scores. In addition, we also performed bootstrapping (see the following content) on the DNN-based classifier to obtain statistical robustness.

Several models, including ridge regression classifier (RR, scikit-learn RidgeClassifier), random forest (RF, scikit-learn RandomForestClassifier), linear support vector machine (LSVM, scikit-learn LinearSVC), gradient boosting (GB, scikit-learn GradientBoostingClassifier) and a dense neural network (DNN, built in keras) were implemented to manifest the classification. RR, RF, LSVM, and GB were conducted using default hyperparameters, except for class_weight, which was set to “balanced”. Details of DNN architecture, hyperparameters, and training are provided in **Table S13**.

**Evaluation of the DNN-based classifier using bootstrapping**

As a final model evaluation, we performed bootstrapping to generate 100 separate train-test splits, re-trained the model on each one, recorded individual F1 scores, and plotted the distribution of recorded F1 scores (**Figures 2E** and **S6**).

**SUPPORTING FIGURES
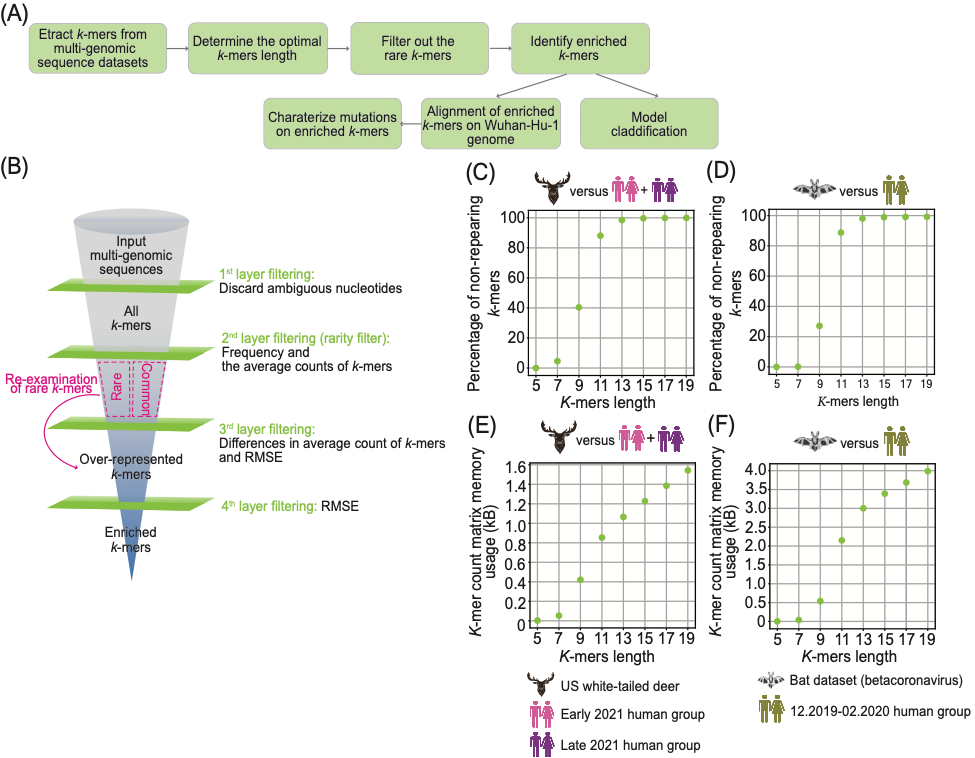
**

**Figure S1.** **Rational design of PORT-EK and determination of the enriched *k*-mers.** (A) The analytical pipeline of PORT-EK. PORT-EK consists of four steps including (1) *k*-mers matrix preparation, (2) *k*-mers filtering and selection, (3) the identification of host-specific mutations, and (4) the classification of hosts. Details are described in the main text. (B) Funnel plot representing the filtering strategies for the selection of enriched *k*-mers. Four layers of filtering were implemented in the PORT-EK pipeline. (C, D) Dot plots representing the percentage of non-repeating *k*-mers based on the percentage of the *k*-mers passing through the rarity filter in the deer (C) and bat (D) datasets, respectively. (E, F) Dot plots representing the memory usage of *k*-mer count matrices (kilobytes, kB) based on the percentage of the *k*-mers passing through the rarity filter in the deer (E) and bat (F) datasets to determine the optimal length of *k*-mers with affordable computing time and memory size. Overall, the *k*-mers of 15 nt in length were chosen for this work.


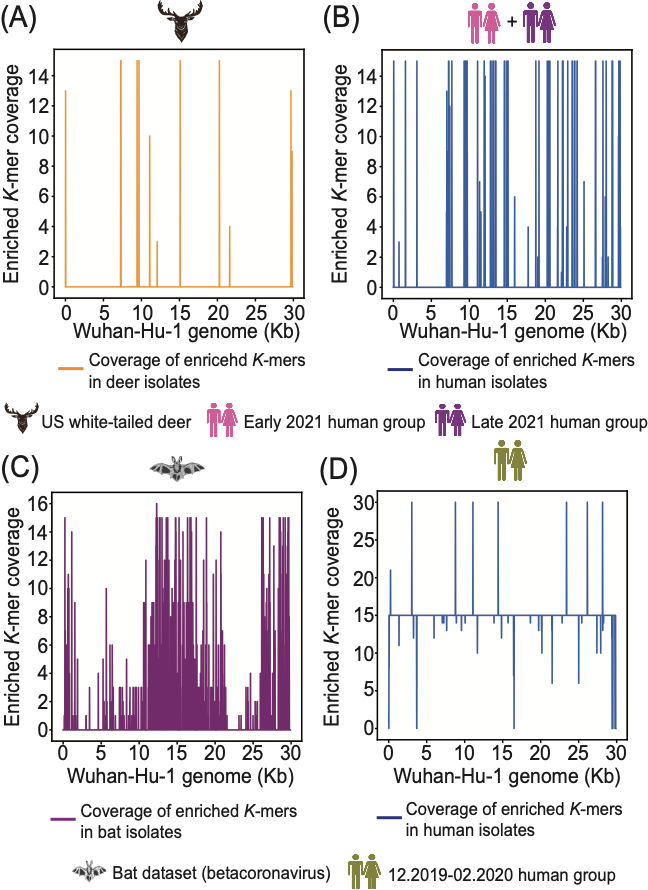


**Figure S2.** **Coverage landscapes of enriched *k*-mers throughout the whole SARS-CoV-2 genome.** (A, B, C, D) Line plots representing the coverage of the SARS-CoV-2 genome per locus overlaid with enriched *k*-mers identified in deer (A) and human (B) isolates collected in the deer dataset or bat (C) and human (D) isolates collected in the bat dataset.


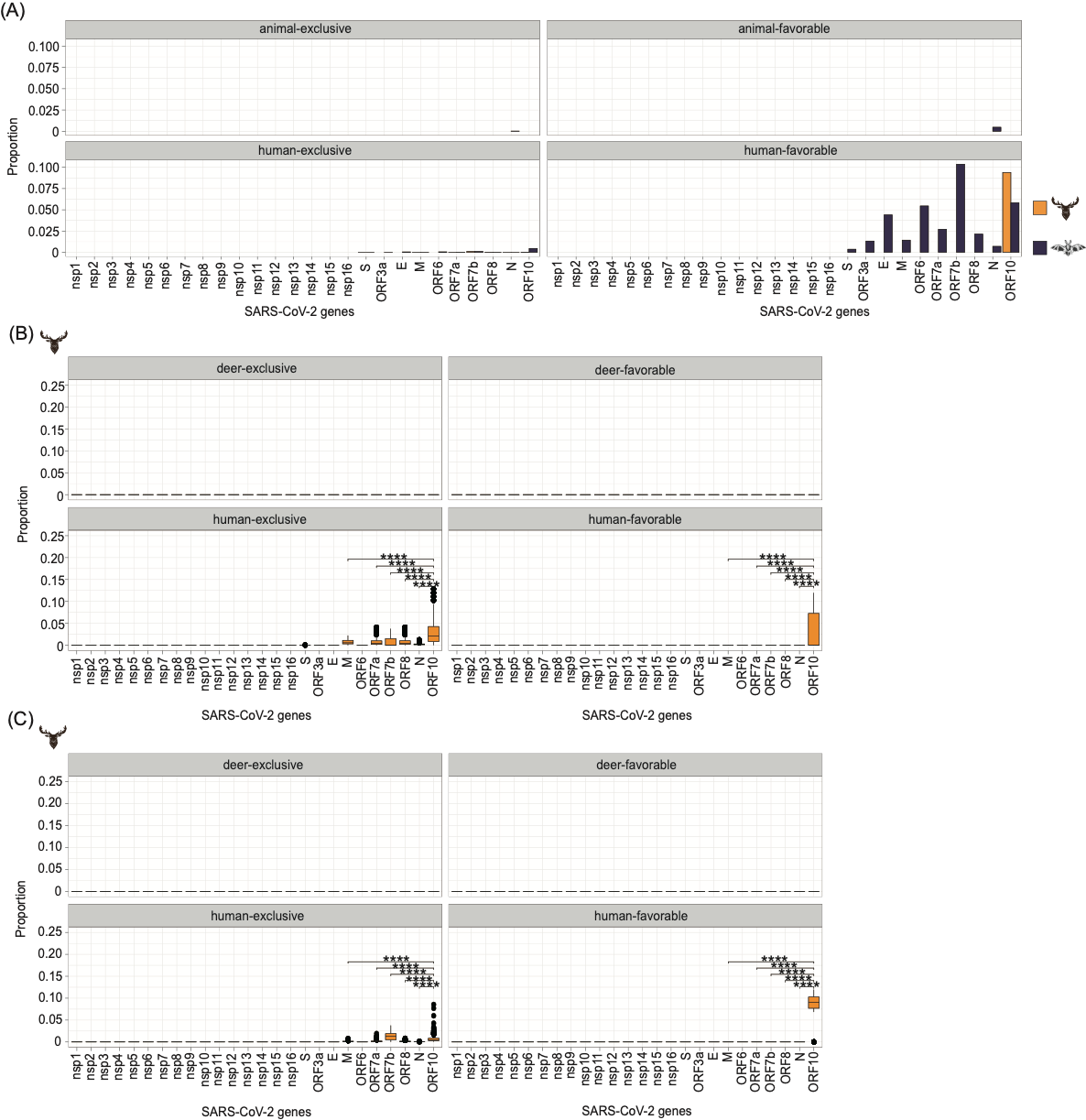


(See caption on page 18)

(See caption on page 18)
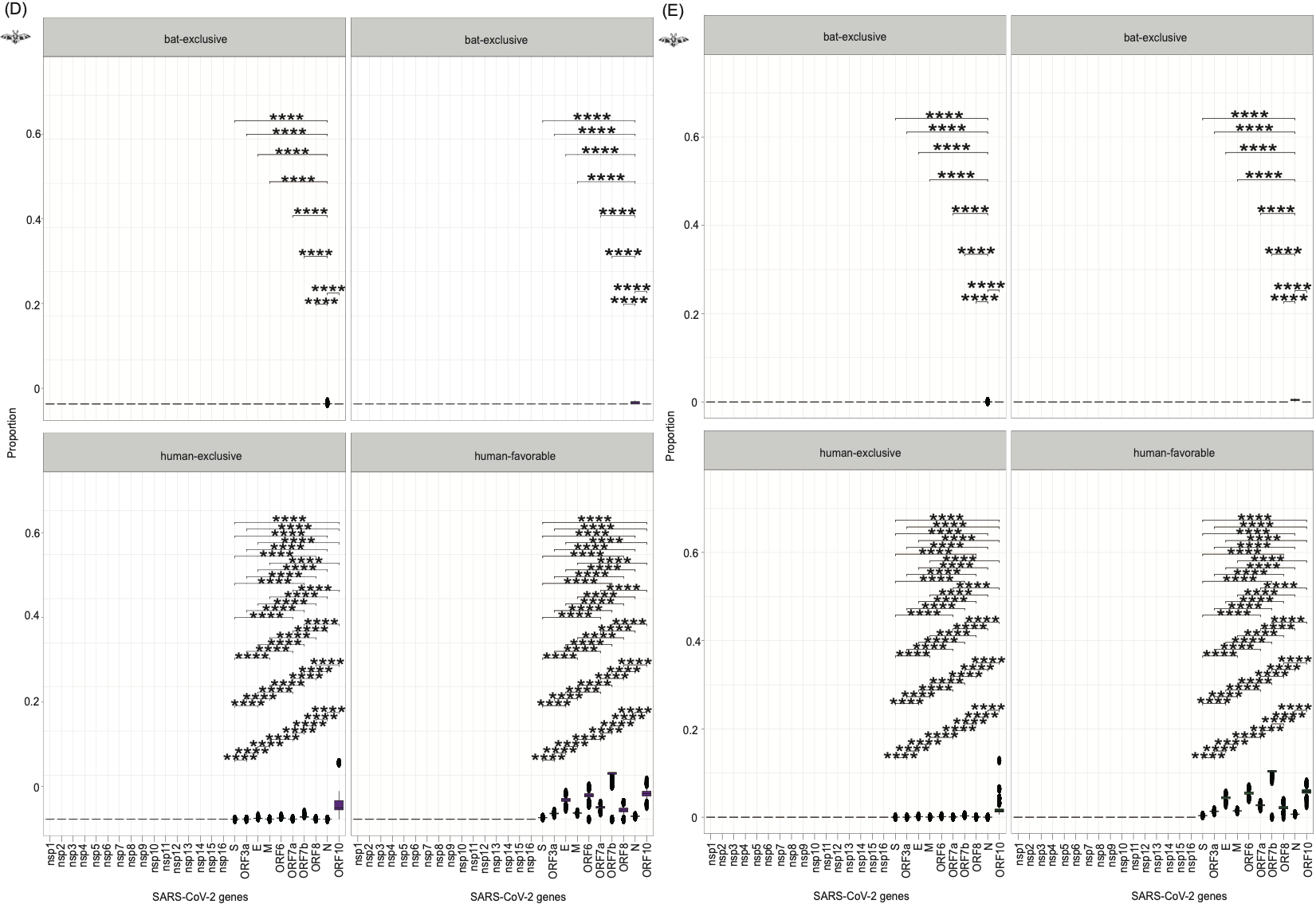


Figure S3. Landscapes of enriched *k*-mers across the SARS-CoV-2 genome. (A) Grouped bar charts representing the enrichment of enriched *k*-mers identified in the deer (orange bars) and bat (purple bars) datasets per SARS-CoV-2 gene. The calculation of the enrichment is described in Supporting Information. Four facets are separated based on the intrinsic property of a genomic locus designated as animal-exclusive loci or animal-favorable loci as well as human-exclusive or human-favorable loci between the deer (orange bars) and bat (purple bars) datasets. Animal-exclusive: the SARS-CoV-2 genomic loci overlaid exclusively with enriched *k*-mers identified in deer (orange bars) or bat (purple bars) isolates; animal-favorable: the loci overlaid enriched *k*-mers present in both host species and are more quantitatively dominant in animal species (deer, orange bars; bats, purple bars) isolates; human-exclusive: the SARS-CoV-2 genomic loci overlaid exclusively with enriched *k*-mers identified in human isolates collected in the deer (orange bars) or bat (purple bars) dataset; human-favorable: the SARS-CoV-2 genomic loci overlaid enriched *k*-mers present in both host species and are more quantitatively dominant in humans (the deer dataset, orange bars; the bat dataset, purple bars). (B, C, D, E) Box plots representing the enrichment of *k*-mers overlaid with SARS-CoV-2 genes. 100 (B) and 500 (C) enriched *k*-mers in the deer dataset as well as 5,000 (D) and 10,000 (E) enriched *k*-mers in the bat dataset were bootstrapped with replacement and this process was repeated 5,000 times. Four facets are separated based on the intrinsic property of a genomic locus designated as deer-exclusive, deer-favorable, human-exclusive, and human-favorable from the isolated collected in the deer dataset (B, C) as well as bat-exclusive, bat-favorable, human-exclusive, and human-favorable from the isolated collected in the bat dataset (D, E). Deer- (B, C) or bat- (D, E) exclusive: the SARS-CoV-2 genomic loci overlaid exclusively with enriched *k*-mers identified in deer (orange bars) or bat (purple bars) isolates; deer- (B, C) or bat- (D, E) favorable: the loci overlaid enriched *k*-mers present in both host species and are more quantitatively dominant in animal species in deer (orange bars) or bat (purple bars) isolates; human-exclusive: the SARS-CoV-2 genomic loci overlaid exclusively with enriched *k*-mers identified in human isolates collected in the deer (B, C: orange bars) or bat (D, E: purple bars) dataset; human-favorable: the SARS-CoV-2 genomic loci overlaid enriched *k*-mers present in both host species and are more quantitatively dominant in humans (the deer dataset, orange bars; the bat dataset, purple bars). Statistical significance was determined using the Wilcoxon test in R with default options. Significance levels are denoted as follows: * *p* 0.05, ** *p* 0.01, *** *p* 0.001, **** *p* 0.0001.


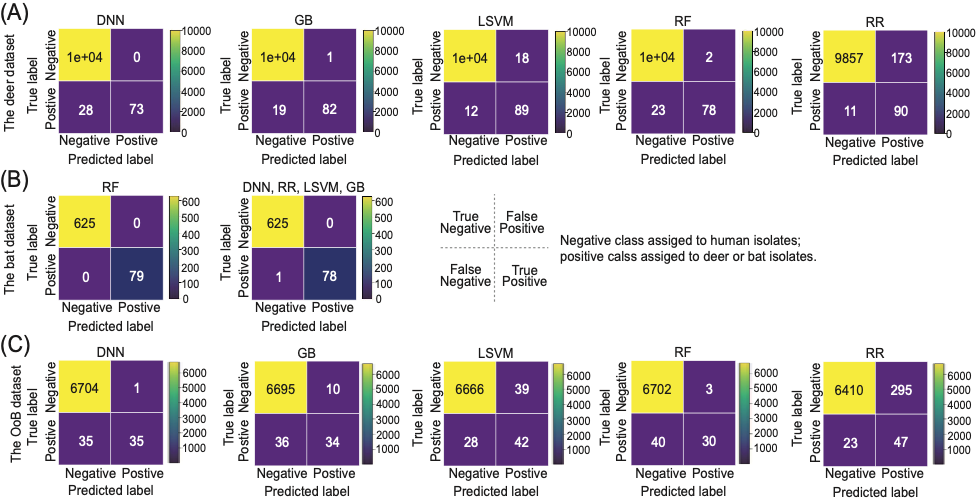


**Figure S4. Prediction summary of the robustness of testing classifiers subjected to different model architectures.** Confusion matrices summarizing the number of predictions that are true negative (top corner on the left-hand side), true positive (bottom corner on the right-hand side), false negative (bottom corner on the left-hand side), and false positive (top corner on the right-hand side). The negative class is designated to individual SARS-CoV-2 genomes predicted as human isolates; the positive class is designated to individual SARS-CoV-2 genomes predicted as animal (deer or bats) isolates. Classifiers were constructed based on the total count of enriched *k*-mers and subjected to the following models, dense neural network (DNN), gradient boosting (GB), linear support vector machine (LSVM), random forest (RF), ridge regression (RR). We applied them to predict the most probable host species of individual SARS-CoV-2 genomic sequences collected in the deer (A), bat (B), and OoB datasets (C).


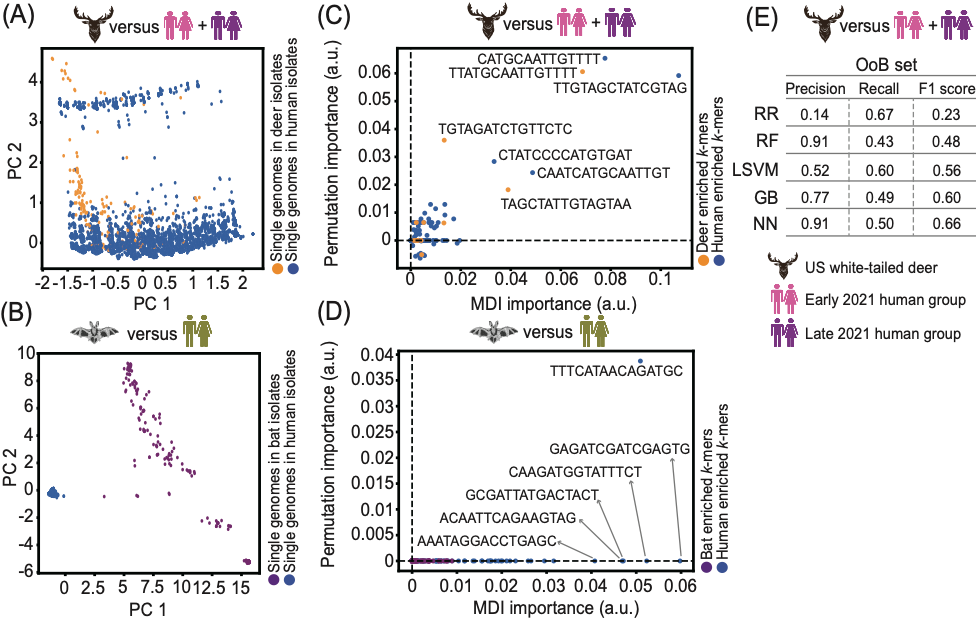
**Figure S5.** **Classification and prediction of the likelihood of SARS-CoV-2 host species based on the enriched *k*-mers count.** (A, B) Two dimensional principal component analysis (PCA) revealing the discrepancy of genomic sequences of isolates collected in the deer (A) and bat (B) datasets. Each dot represents a single genome of an isolate. Dots marked in orange indicate genomic sequences from deer isolates (A); dots marked in purple indicate genomic sequences from bat isolates (B); dots marked in blue indicate genomic sequences from human isolates (A, B). (C, D) Scatter plots unveiling the critical *k*-mer sequences for the classification of the likelihood of SARS-CoV-2 host species: deer versus humans (C) and bats versus humans (D). Based on the measures of the mean decrease of impurity (MDI) feature importance and permutation-based importance, a random forest model was constructed for classification. A total of seven (C) and six (D) enriched *k*-mers essential for the classification of the likelihood of SARS-CoV-2 host species were identified. (E) A table recording the record of F1 scores computed by the OoB dataset. Five models, including ridge regression classifier (RR), random forest (RF), linear support vector machine (LSVM), gradient boosting (GB), and a dense neural network (DNN) were implemented to verify the effectiveness of each model.


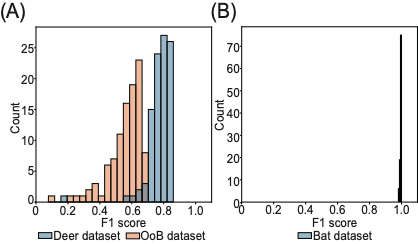


**Figure S6. Bootstrapping on the subsets of multi-genomes in testing datasets.** (A, B) Histogram representing the distribution of F1 scores measured from 100 independent train-test splits on single genomes of isolates collected in the deer, OoB (A), and bat (B) datasets.

(See caption on page 24)
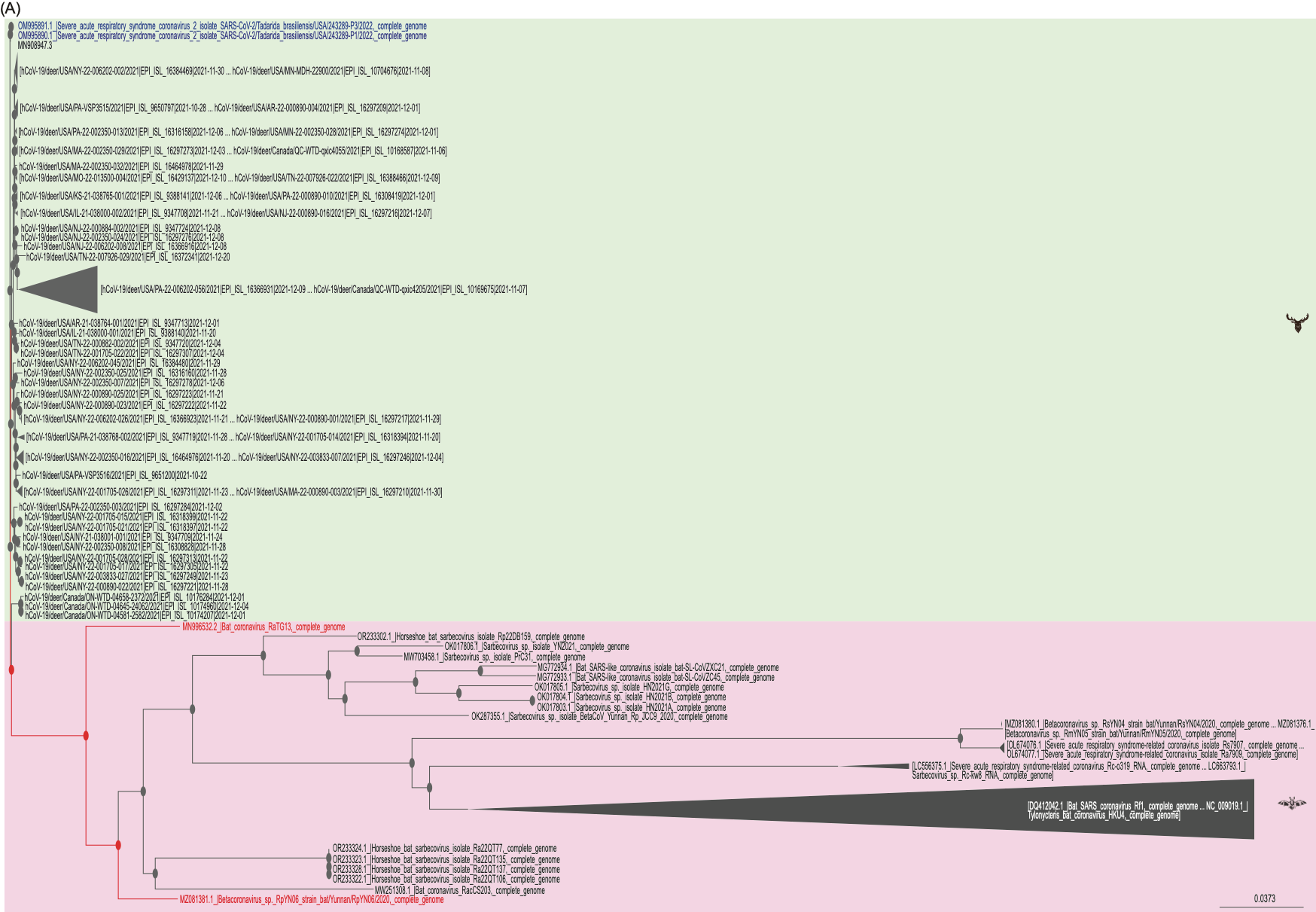


(See caption on page 24)
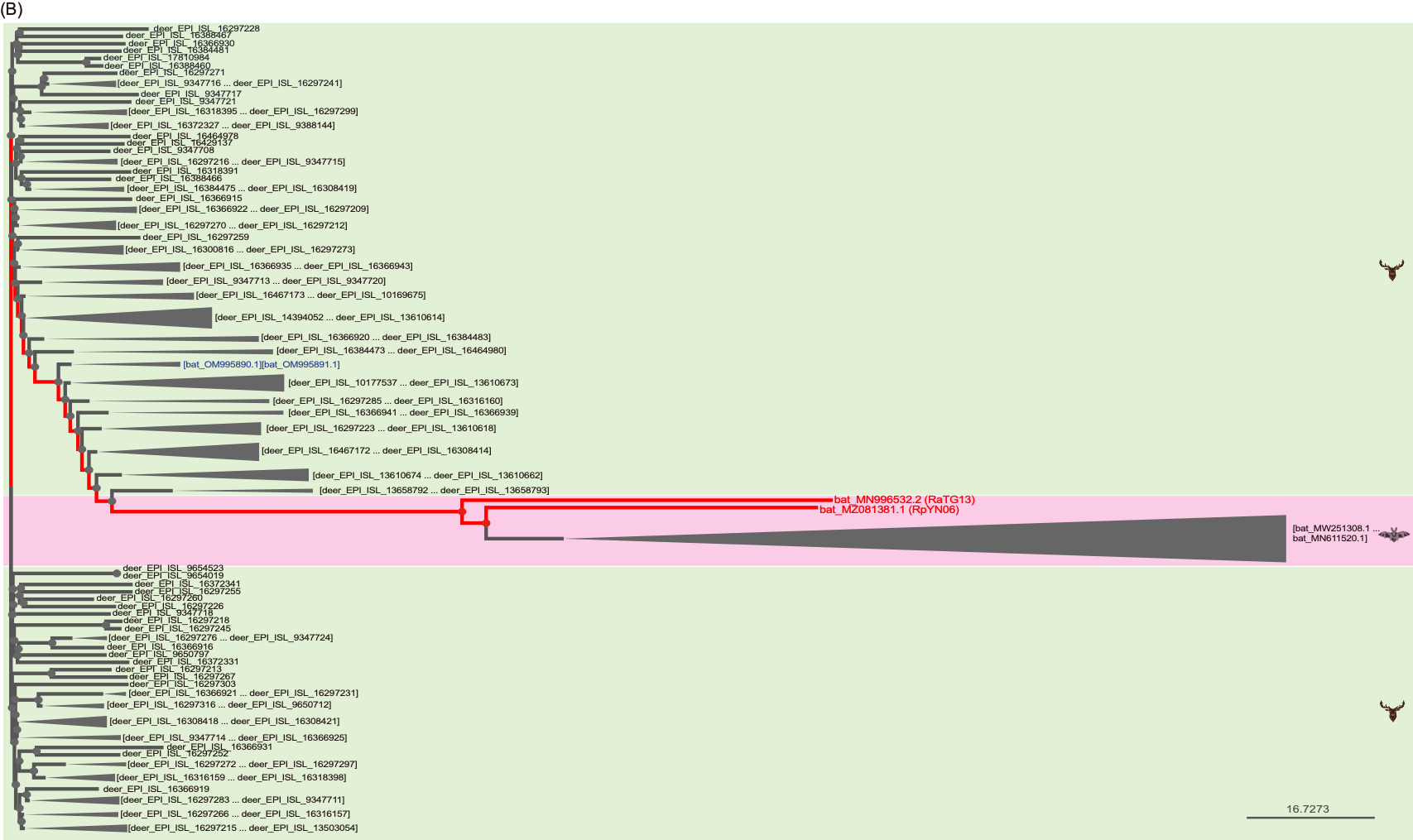


Figure S7. Phylogenetic analysis of SARS-CoV-2 isolates from US white-tailed deer and bats. Extended versions of phylogenetic trees were constructed using either complete genomic sequences (A) or the enriched *k*-mer count (B) between two animal species, isolated from US white-tailed deer and bat reservoirs, consisting of 34 bat species (Table S1). Branches, branch points, and isolates written in red indicate the SARS-CoV-2 species, RaTG13 and RpYN06, which are two bat coronaviruses genetically close to deer SARS-CoV-2. Isolates written in blue indicate two bat isolates, OM995890.1 and OM995891.1, with the uncertainty of their origins from wild bats. Branches with a green background correspond to deer isolates, whereas branches with a pink background correspond to bat isolates. Compressed versions of phylogenetic trees are provided in Figures 2F and 2G.

**SUPPORTING FILES**

**File S1.** Deer dataset: US white-tailed deer group (*Odocoileus virginianus*) - EPI_SET_240422va, <https://doi.org/10.55876/gis8.240422va>. GISIAD supplemental table for deer coronavirus sequences of deer dataset.


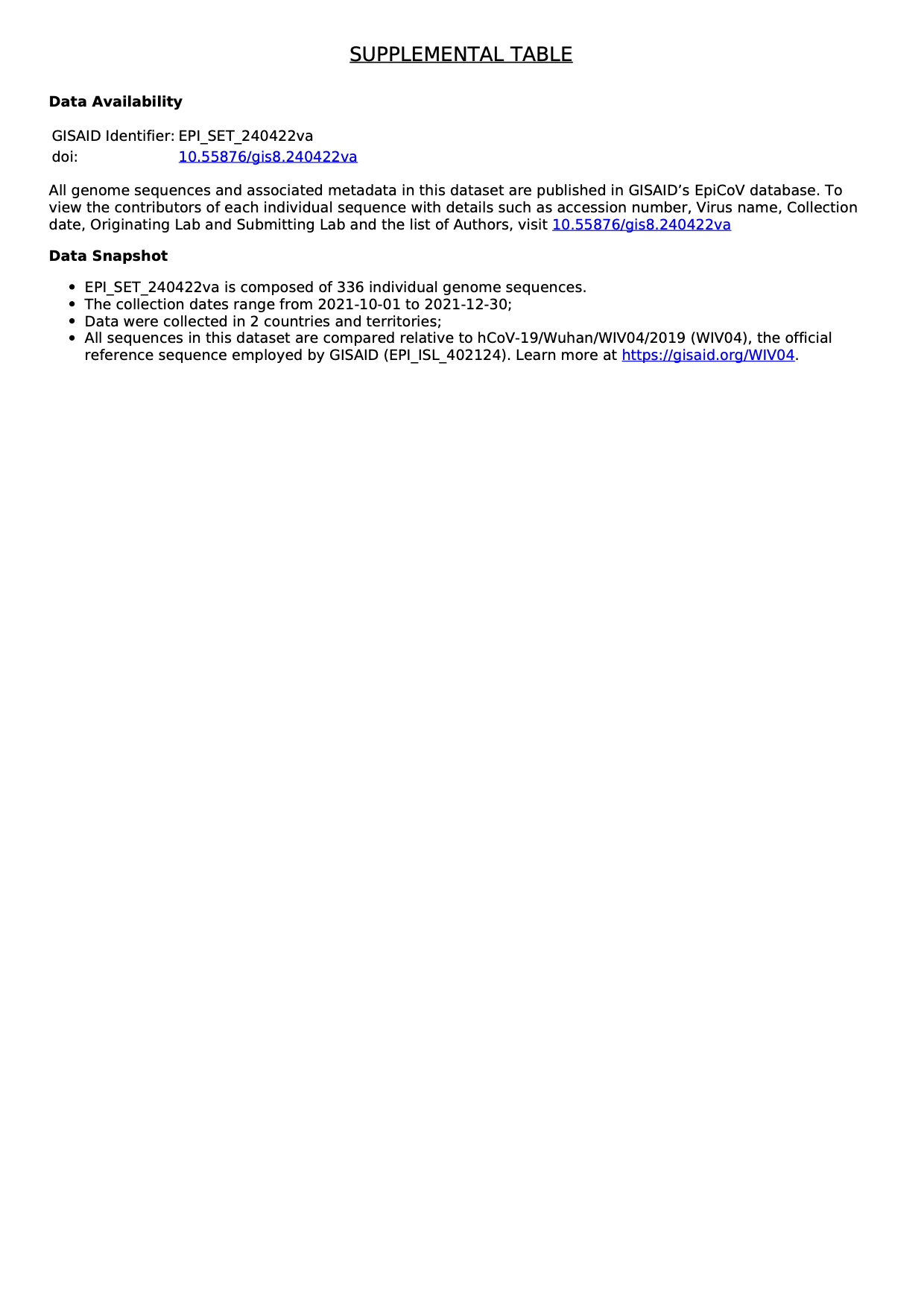


**File S2.** Deer dataset: early 2021 human group (April 2021) - EPI_SET_240422rw, <https://doi.org/10.55876/gis8.240422rw>. GISIAD supplemental table for early 2021 human coronavirus sequences of deer dataset.


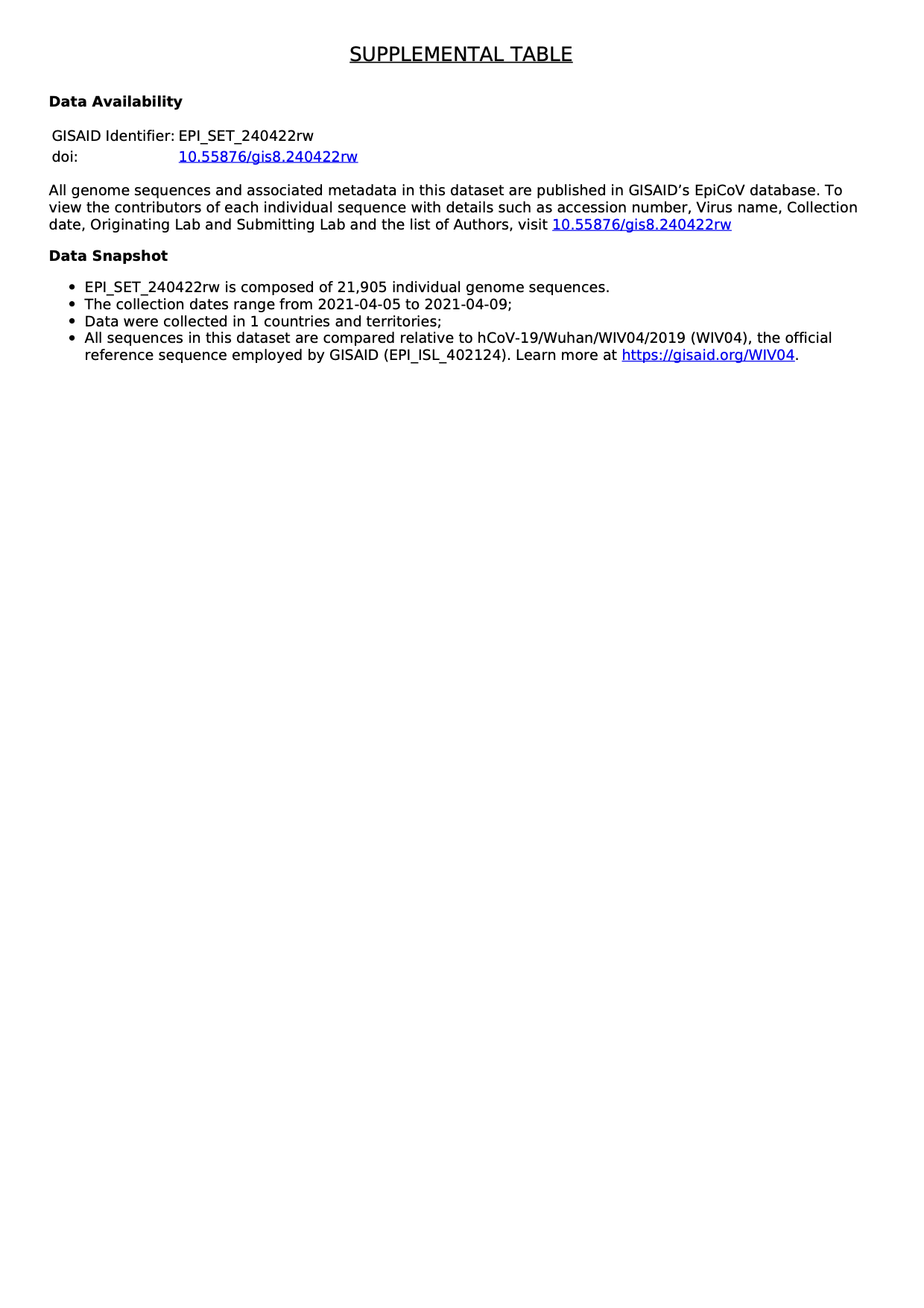


**File S3.** Deer set: late 2021 human group (November 2021) - EPI_SET_240422qc,<https://doi.org/10.55876/gis8.240422qc>. GISIAD supplemental table for late 2021 human coronavirus sequences of deer dataset.


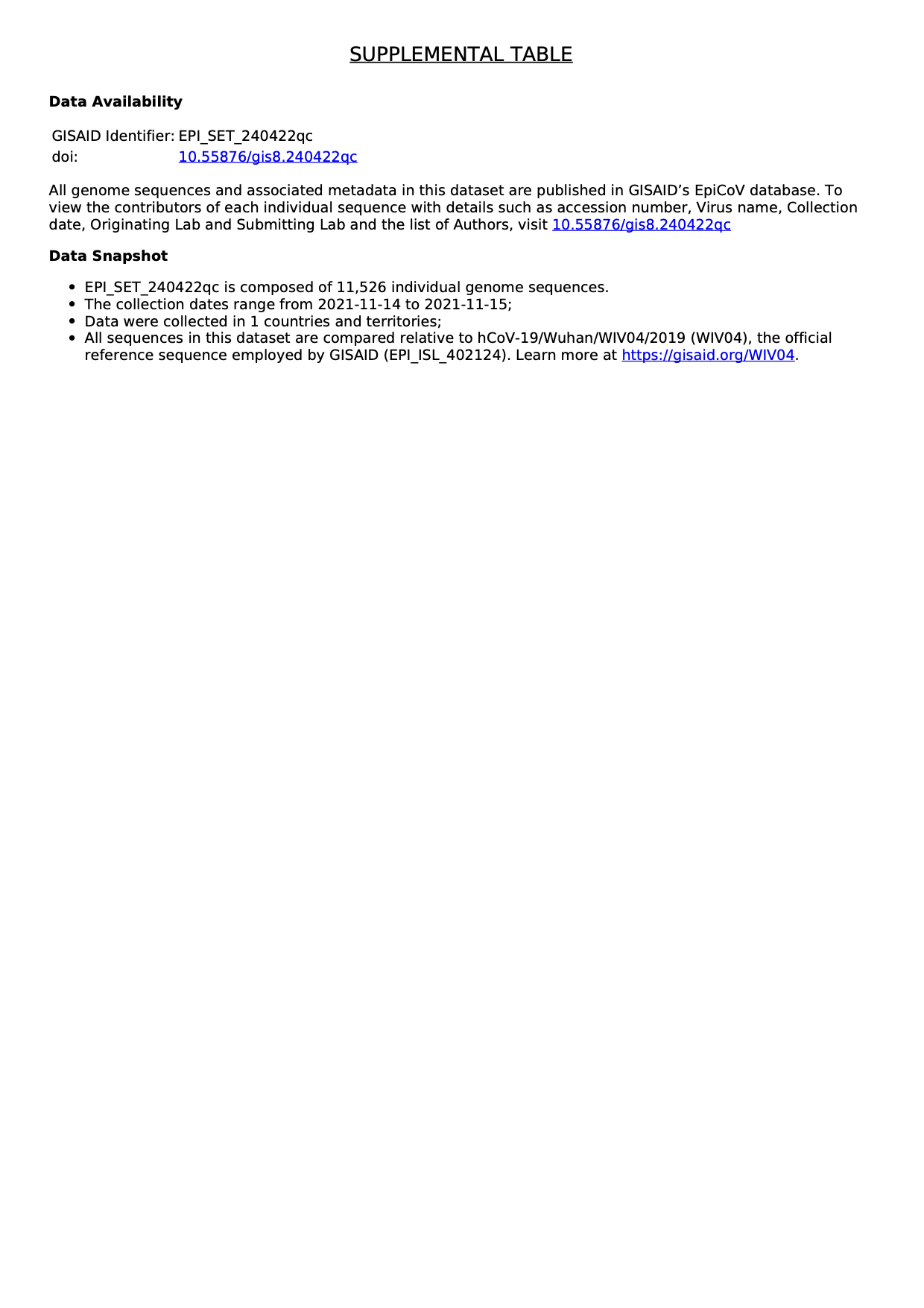


**File S4.** OoB dataset: US white-tailed deer group (*Odocoileus virginianus*) - EPI_SET_240422oy,<https://doi.org/10.55876/gis8.240422oy>. GISIAD supplemental table for deer coronavirus sequences of OoB dataset.


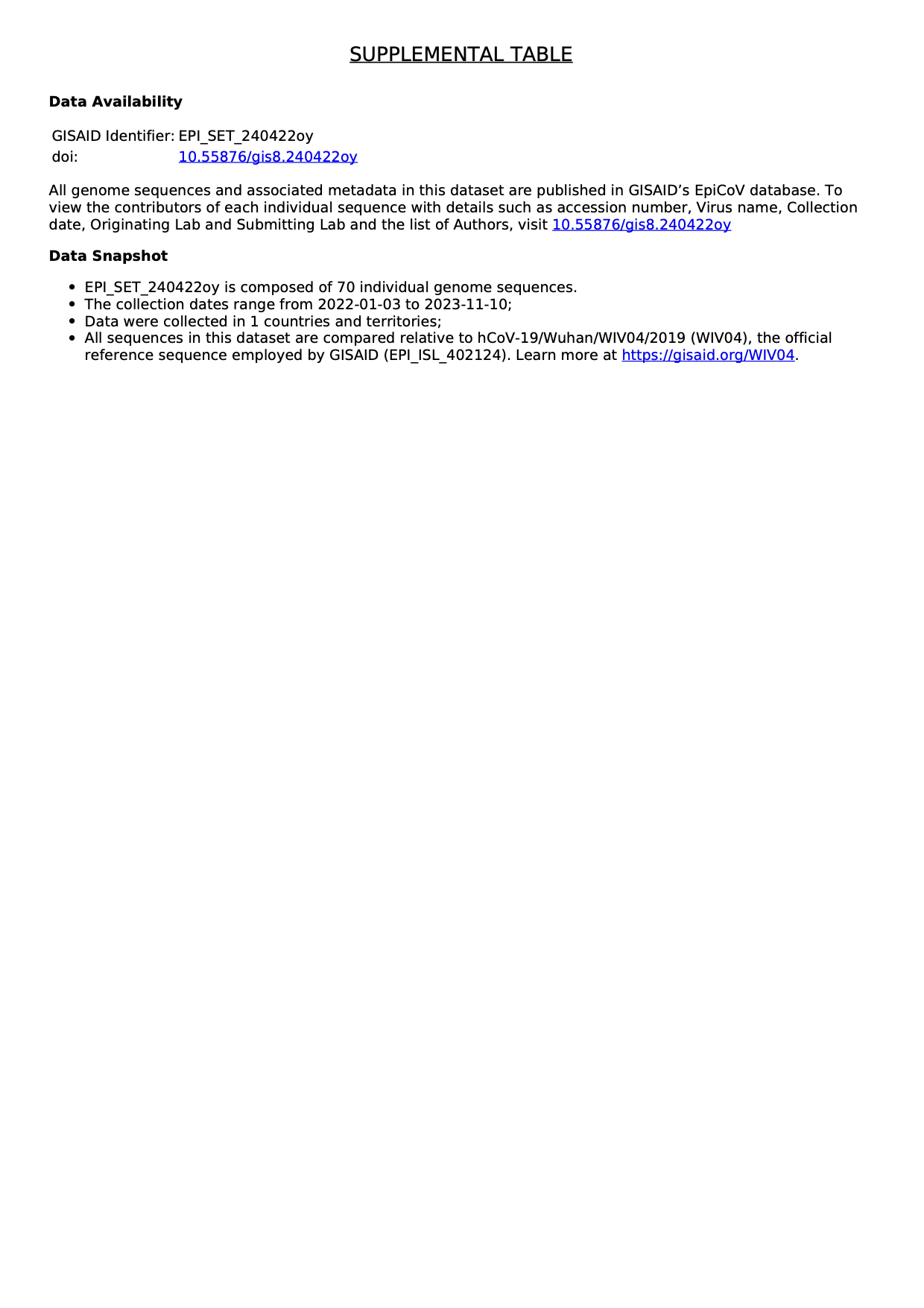


**File S5.** OoB dataset: human group - EPI_SET_240422xu, <https://doi.org/10.55876/gis8.240422xu>. GISIAD supplemental table for human coronavirus sequences of OoB dataset.


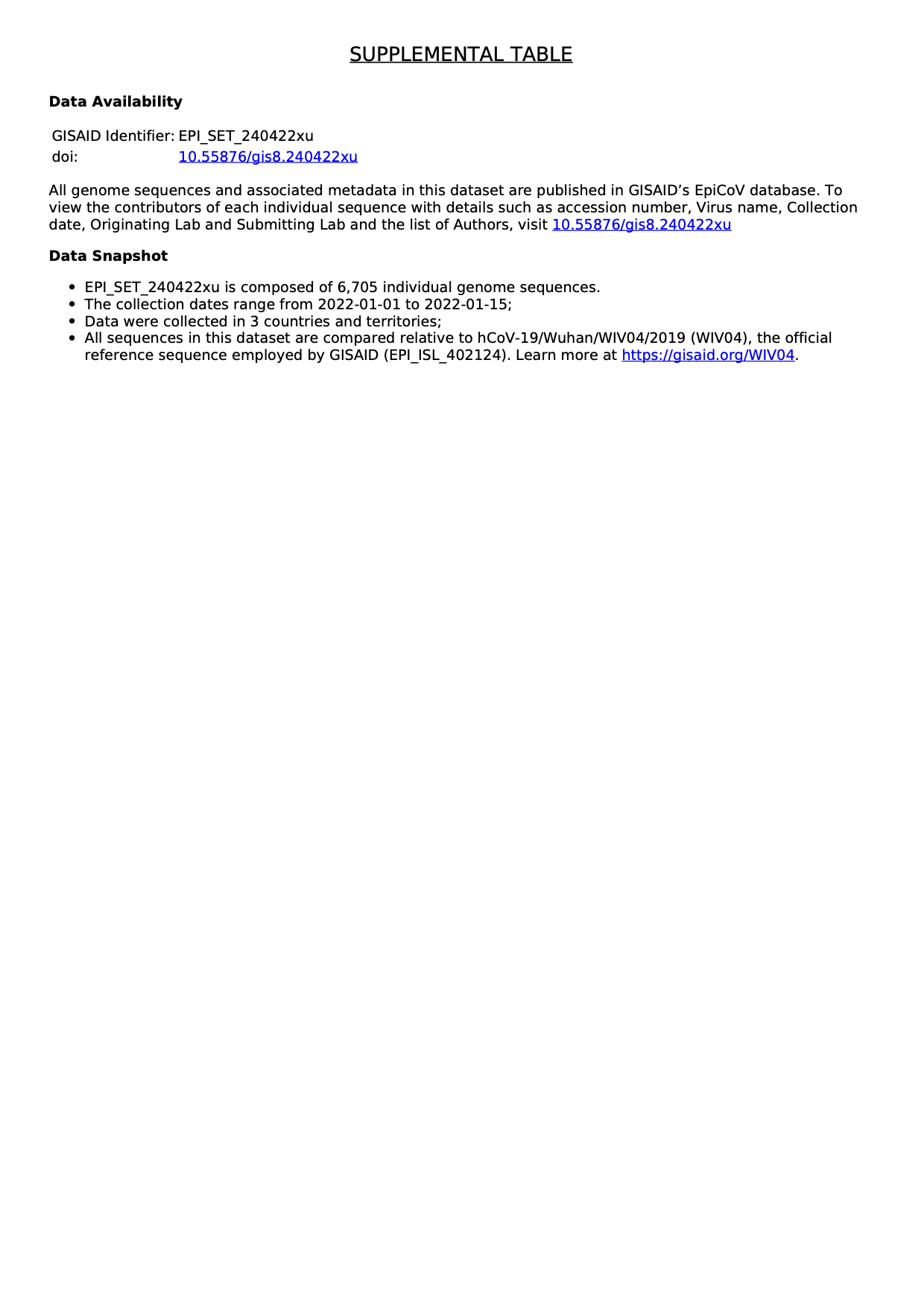


**File S6.** Bat dataset, human group - EPI_SET_240422qm, <https://doi.org/10.55876/gis8.240422qm>. GISIAD supplemental table for human coronavirus sequences of bat dataset.


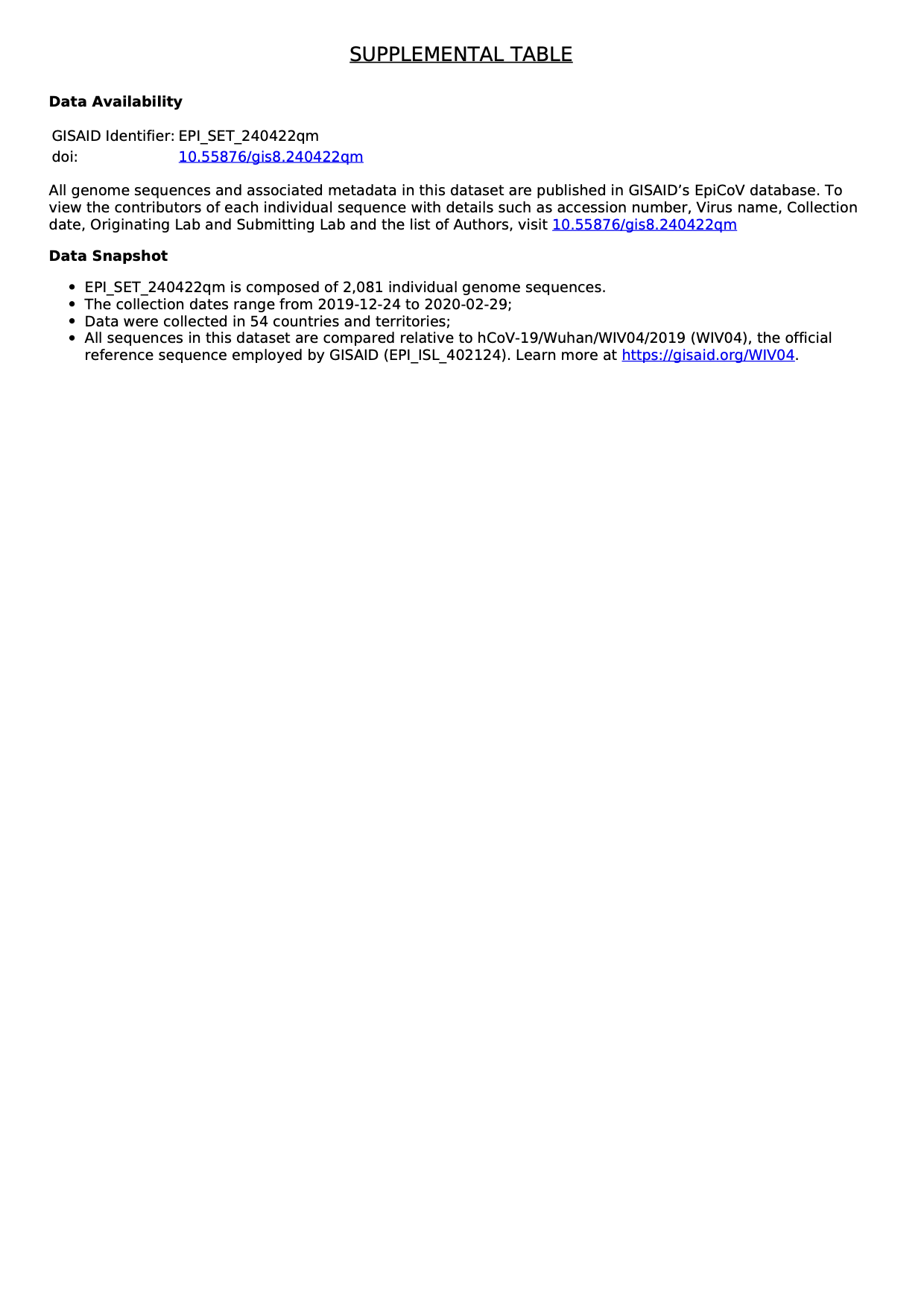


**REFERENCES**

1. Liu Zeyi, Yang Ma, Qing Cheng, Zhoung Liu. 2022. “Finding Asymptomatic Spreaders in a COVID-19 Transmission Network by Graph Attention Networks.” *Viruses* 14. <https://doi.org/10.3390/v14081659>

2. Singh Rohan, Sunil Nagpal, Nishal K. Pinna, Sharmila S. Mande. 2022. “Tracking mutational semantics of SARS-CoV-2 genomes.” *Sci Rep* 12: 15704. <https://doi.org/10.1038/s41598-022-20000-5>

3. Singh Bist Prem, Hilal Tayara, Kil To Chong. 2023. “Sars-escape network for escape prediction of SARS-COV-2.” *Brief Bioinform* 24. <https://doi.org/10.1093/bib/bbad140>

4. Tong Yuantao, Fanglin Tan, Honglian Huang, Zeyu Zhang, Hui Zong, Yujia Xie, Danqi Huang, et al. 2023. “ViMRT: a text-mining tool and search engine for automated virus mutation recognition.” *Bioinformatics* 39. <https://doi.org/10.1093/bioinformatics/btac721>

5. Zhou Binbin, Hang Zhou, Xue Zhang, Xiaobin Xu, Yi Chai, Zengwei Zheng, Alex Chichung Kot, Zhan Zhou. 2023. “TEMPO: A transformer-based mutation prediction framework for SARS-CoV-2 evolution.” *Comput Biol Med* 152: 106264. <https://doi.org/10.1016/j.compbiomed.2022.106264>

6. Kirk M. Jessime, Susan O. Kim, Kaoru Inoue, Matthew J. Smola, David M. Lee, Megan D, Schertzer, Joshua S. Wooten, et al. 2018. “Functional classification of long non-coding RNAs by k-mer content.” *Nat Genet* 50: 1474–1482. <https://doi.org/10.1038/s41588-018-0207-8>

7. Lorenzi Claudio, Sylvain Barriere, Jean-Philippe Villemin, Laureline Dejardin Bretones, Alban Mancheron, William Ritchie. 2020. “iMOKA: k-mer based software to analyze large collections of sequencing data.” *Genome Biol* 21: 261. <https://doi.org/10.1186/s13059-020-02165-2>

8. Forsdyke R Donald. 2019. “Success of alignment-free oligonucleotide (k-mer) analysis confirms relative importance of genomes not genes in speciation and phylogeny.” *Biol J Linn Soc Lond* 128: 239-250 <https://doi.org/10.1093/biolinnean/blz096>

9. Li Wentian, Jerome Freudenberg, Jan Freudenberg. 2019. “Alignment-free approaches for predicting novel Nuclear Mitochondrial Segments (NUMTs) in the human genome.” *Gene* 691: 141–152. <https://doi.org/10.1016/j.gene.2018.12.040>

10. Ma Yuanlin, Zuguo Yu, Runbin Tang, Xianhua Xie, Guosheng Han, Vo V. Anh. 2020. “Phylogenetic Analysis of HIV-1 Genomes Based on the Position-Weighted K-mers Method.” *Entropy (Basel)* 22. <https://doi.org/10.3390/e22020255>

11. Wen Jia, YuYan Zhang, Stephen S.T. Yau. 2014. “k-mer sparse matrix model for genetic sequence and its applications in sequence comparison.” *J Theor Biol* 363: 145–150. <https://doi.org/10.1016/j.jtbi.2014.08.028>

12. Pimentel A.F. Marco, David A. Clifton, Lei Clifton, Lionel Tarassenko. 2014. “A review of novelty detection.” *Signal Processing* 99: 215–249. <https://doi.org/10.1016/j.sigpro.2013.12.026>

13. Zimek Arthur, Peter Filzmoser. 2018. “There and back again: Outlier detection between statistical reasoning and data mining algorithms.” *Wiley Interdiscip Rev Data Min Knowl Discov* 8: e1280. <https://doi.org/10.1002/widm.1280>

14. Nassif B. Ali, Manar A. Talib, Qassin Nasir, Fatima M. Dakalbab. 2021. “Machine learning for anomaly detection: A systematic review.” *IEEE Access* 9: 78658–78700. <https://doi.org/10.1109/access.2021.3083060>

15. Alsulimani Ahmad, Naseem Akhter, Fatima Jameela, Rnda I. Ashgar, Arshad Jawed, Mohammed A. Hassani, Sajad A. Dar. 2024. “The Impact of Artificial Intelligence on Microbial Diagnosis.” *Microorganisms* 12. <https://doi.org/10.3390/microorganisms12061051>

16. Nordström J. V. Kar, Maria C. Albani, Geo V. James, Caroline Gutjahr, Benjamin Hartwig, Franziska Turck, Uta Paszkowski, George Coupland, Korbinian Schneeberger. 2013. “Mutation identification by direct comparison of whole-genome sequencing data from mutant and wild-type individuals using k-mers.” *Nat Biotechnol* 31: 325–330. <https://doi.org/10.1038/nbt.2515>

17. Bray L. Nicolas, Harold Pimentel, Páll Melsted, Lior Pachter. 2016. “Near-optimal probabilistic RNA-seq quantification.” *Nat Biotechnol* 34: 525–527. <https://doi.org/10.1038/nbt.3519>

18. Ondov D. Brian, Todd J. Treangen, Páll Melsted, Adam B. Mallonee, Nicholas H. Bergma, Sergey Koren, Adam M. Phillippy. 2016. “Mash: fast genome and metagenome distance estimation using MinHash.” *Genome Biol* 17: 132. <https://doi.org/10.1186/s13059-016-0997-x>

19. Shajii Ariya, Deniz Yorukoglu, Yun W. Yu, Bonnie Berger. 2016. “Fast genotyping of known SNPs through approximate k-mer matching.” *Bioinformatics* 32: i538–i544. <https://doi.org/10.1093/bioinformatics/btw460>

20. Patro Rob, Geet Duggal, Michael I. Love, Rafael A. Irizarry, Carl Kingsford. 2017. “Salmon provides fast and bias-aware quantification of transcript expression.” *Nat Methods* 14: 417–419. <https://doi.org/10.1038/nmeth.4197>

21. Ounit Rachid, Steve Wanamaker, Timothy J. Close, Stefano Lonardi. 2015. “CLARK: fast and accurate classification of metagenomic and genomic sequences using discriminative k-mers.” *BMC Genomics* 16: 236. <https://doi.org/10.1186/s12864-015-1419-2>

22. Audoux Jérôme, Nicolas Philippe, Rayan Chikhi, Mikaël Salson, Mélina Gallopin, Marc Gabriel, Jérémy Le Coz, Emilie Drouineau, Thérèse Commes, Daniel Gautheret. 2017. “DE-kupl: exhaustive capture of biological variation in RNA-seq data through k-mer decomposition.” *Genome Biol* 18: 243. <https://doi.org/10.1186/s13059-017-1372-2>

23. Lau T. Billy, Dmitri Pavlichin, Anna C. Hooker, Alison Almeda, Giwon Shin, Jiamin Chen, Malaya K. Sahoo, et al. 2021. “Profiling SARS-CoV-2 mutation fingerprints that range from the viral pangenome to individual infection quasispecies.” *Genome Medicine* 13: 1–23. <https://doi.org/10.1186/s13073-021-00882-2>

24. Louppe Gilles. 2014. “Understanding random forests: From theory to practice.” *arXiv [stat.ML]* 1407.7502. <https://doi.org/10.48550/ARXIV.1407.7502>

25. Li Xiao, Yu Wang, Sumanta Basu, Karl Kumbier, Bin Yu. 2019. “A debiased MDI feature importance measure for Random Forests.” *arXiv [stat.ML]* 1906.10845. <https://doi.org/10.48550/ARXIV.1906.10845>

26. Moore H. Jason. 1999. “Bootstrapping, permutation testing and the method of surrogate data.” *Phys Med Biol* 44: L11–2. <https://doi.org/10.1088/0031-9155/44/6/101>

27. Nabhan R. Ahmed, Indra N. Sarkar. 2012. “The impact of taxon sampling on phylogenetic inference: a review of two decades of controversy.” *Brief Bioinform* 13: 122–134. <https://doi.org/10.1093/bib/bbr014>

28. Palmer V. Mitchell, Mathias Martins, Shollie Falkenberg, Alexandra Buckley, Leonardo C. Caserta, Patrick K. Mitchell, Eric D. Cassmann, et al. 2021. “Susceptibility of white-tailed deer (*Odocoileus virginianus*) to SARS-CoV-2.” *J Virol* 95. <https://doi.org/10.1128/JVI.00083-21>

29. Lassmann Timo. 2019. “Kalign 3: multiple sequence alignment of large data sets.” *Bioinformatics* 36: 1928–1929. <https://doi.org/10.1093/bioinformatics/btz795>

30. Cock J. A. Peter, Tiago Antao, Jeffrey T. Chang, Brad A. Chapman, Cymon J. Cox, Andrew Dalke, Iddo Friedberg, et al. 2009. “Biopython: freely available Python tools for computational molecular biology and bioinformatics.” *Bioinformatics* 25: 1422–1423. <https://doi.org/10.1093/bioinformatics/btp163>

31. Virtanen Pauli, Ralf Gommers, Travis E. Oliphant, Matt Haberland, Tyler Reddy, David Cournapeau, Evgeni Burovski, et al. 2020. “SciPy 1.0: fundamental algorithms for scientific computing in Python.” *Nat Methods* 17: 261–272. <https://doi.org/10.1038/s41592-019-0686-2>

32. Saitou Naruya, Masatoshi Nei. 1987. “The neighbor-joining method: a new method for reconstructing phylogenetic trees.” *Mol Biol Evol* 4: 406–425. <https://doi.org/10.1093/oxfordjournals.molbev.a040454>

33. The pandas development team. 2024. “pandas-dev/pandas: Pandas.” *Zenodo* <https://doi.org/10.5281/ZENODO.3509134>

34. Altmann André, Laura Toloşi, Oliver Sander, Thomas Lengauer. 2010. “Permutation importance: a corrected feature importance measure.” *Bioinformatics* 26: 1340–1347. <https://doi.org/10.1093/bioinformatics/btq134>

35. Wang Ying, Qi Chen, Chao Deng, Yiluan Zheng, Fengzhu Sun. 2020. “KmerGO: A Tool to Identify Group-Specific Sequences With *k*-mers.” *Front Microbiol* 11: 2067. <https://doi.org/10.3389/fmicb.2020.02067>
